# Effectiveness and safety of esketamine in laparoscopic surgery patients: a systematic review and meta-analysis of randomized controlled trials

**DOI:** 10.3389/fphar.2025.1663348

**Published:** 2025-10-21

**Authors:** Shuhui Wang, Wei Hao, Hao Fan, Jiasheng Wu, Lifang Wu

**Affiliations:** ^1^ Department of Anesthesiology, Inner Mongolia Medical University Affiliated Hospital, Hohhot, China; ^2^ Department of Anesthesiology, The Fifth Clinical Medical College of Inner Mongolia Medical University, Hohhot, China; ^3^ Department of Anesthesiology, Chifeng Hospital, Chifeng, China

**Keywords:** esketamine, laparoscopic surgery, meta-analysis, randomized controlled trial, perioperative period

## Abstract

**Objectives:**

Laparoscopic surgery is commonly performed, with perioperative treatments aimed at minimizing its impact on patients. Esketamine, known for its antidepressant mechanism, has gained attention as an anesthetic. This review evaluates its effectiveness and safety in laparoscopic surgery patients, since existing trials report conflicting results.

**Patients and methods:**

A systematic search across eight databases identified RCTs (Randomized Controlled Trials) on esketamine’s effects in laparoscopic surgery patients. Outcomes assessed included VAS (Visual Analog Scale), AIS (Athens Insomnia Scale), NRS (Numeric Rating Scale), QoR-15 (Postoperative Quality of Recovery), remifentanil consumption, ICFS-10 (Inpatient Cognitive Function Scale) scores, and plasma BDNF (brain-derived neurotrophic factor) concentrations. The study is registered in PROSPERO (CRD42025630085).

**Results:**

Fifteen studies involving 1,553 participants were included. Esketamine reduced postoperative VAS (SMD: −0.47; 95% CI [-0.89, −0.05]; P = 0.027) and NRS scores (SMD: −0.36; 95% CI [-0.70, −0.01]; P = 0.042). It also decreased AIS scores on the first (SMD: −0.55; 95% CI [-1.03, −0.07]; P = 0.026) and third days (SMD: −0.85; 95% CI [−1.42, −0.29]; P = 0.003), and ICFS-10 scores (first: SMD: −0.55; third: SMD: −0.62). Additionally, esketamine lowered remifentanil consumption (SMD: −0.58; P = 0.003) and infusion rate (SMD: −0.40; P = 0.001), while increasing plasma BDNF concentrations (SMD: 1.19; P = 0.044). Sensitivity analysis confirmed the stability of these results.

**Conclusion:**

Esketamine alleviates postoperative pain, reduces remifentanil and opioid consumption, improves sleep quality and recovery, mitigates postoperative fatigue, and increases plasma BDNF concentrations in laparoscopic surgery patients. Nevertheless, this meta-analysis still has certain limitations, most notably the high heterogeneity of the studies incorporated and the limited geographical coverage of the research sites. Further studies are needed to confirm these findings and support its use in improving perioperative outcomes.

## 1 Introduction

Laparoscopic surgery is becoming more widespread, but pneumoperitoneum can adversely affect the respiratory, circulatory, and nervous systems, leading to postoperative pain and disrupted sleep quality. Despite these challenges, it offers benefits, including lower complication rates, quicker recovery, and minimal iatrogenic trauma (Sjövall et al., 2015; Zhao et al., 2024). Currently, however, very little comprehensive assessment has been conducted on the effectiveness of ketamine in the context of diverse laparoscopic surgical procedures. Furthermore, early pain after laparoscopic procedures may be on par with, or even exceed in severity, that of open surgery—highlighting the urgent need for a robust pain management plan. Recent studies recognize esketamine, a novel N-methyl-D-aspartate (NMDA) receptor antagonist with greater affinity than ketamine, as the more potent S-enantiomer of racemic ketamine, which is approved for use in several countries, including China (Xu Y. et al., 2023; Hovaguimian et al., 2018; Guo et al., 2023; Xie et al., 2023; Chen et al., 2024). Esketamine possesses not only a distinct antidepressant mechanism but also the capacity to potentiate the effects of anesthesia and analgesia. It has a five-hour elimination half-life, near 100% intravenous bioavailability, and 30%–50% intranasal bioavailability (d'Andrea et al., 2025; Di Nicola et al., 2025; Pettorruso et al., 2023; Sarasso et al., 2024). However, the dose equivalence between intravenous ketamine and intranasal esketamine remains undefined (McIntyre et al., 2021). Esketamine is approved by the U.S. Food and Drug Administration for the treatment of depression (McIntyre et al., 2023). Initially recognized for its distinctive antidepressant mechanism, ketamine has recently found widespread application in anesthesia and postoperative analgesia. Emerging studies have further demonstrated that ketamine also holds promising potential in facilitating postoperative recovery, with benefits including emotional stabilization and cognitive function protection. Notably, existing clinical trials have similarly indicated that when this drug is used for postoperative analgesia and antidepressant treatment, certain contradictions emerge in practical application, posing unresolved issues for subsequent clinical medication and mechanism research. This meta-analysis represents the first study to systematically assess the efficacy and safety of ketamine administration across diverse laparoscopic surgical procedures. It comprehensively elaborates on ketamine’s perioperative impact on patients, establishes an evidence-based medicine (EBM) framework to guide the development of clinical diagnostic and treatment protocols, and provides evidence-backed support for advancing the progress of EBM-related research in this field.

## 2 Methods

This Preferred Reporting Items for Systematic Reviews and Meta-Analyses (PRISMA)-compliant meta-analysis (Page et al., 2021) was prospectively registered in PROSPERO (CRD42025630085).

### 2.1 Search strategy

A systematic search of multiple databases identified studies assessing intravenous esketamine efficacy in laparoscopic patients. Systematic database searches (PubMed, Embase, Cochrane Library, Web of Science, China National Knowledge Infrastructure (CNKI), VIP Database, Wanfang, and SinoMed) incorporated Boolean logic with MeSH/keyword pairs (“ketamine,” “esketamine,” “laparoscopic surgery,” “laparoscopy”). Dual-layer reference screening (retrieved articles, study citations) ensured that no relevant literature was omitted. Full search protocols were presented in Supplementary Table S1.

### 2.2 Inclusion and exclusion criteria

Study selection adhered to the Participants, Intervention, Comparison, Outcomes, Study Design (PICOS) framework (Munn et al., 2018):

Participants: patients undergoing diverse laparoscopic procedures under general anesthesia;

Intervention: perioperative intravenous esketamine without dose/timing restrictions;

Comparison: non-esketamine controls;

Outcomes: primary (pre-/postoperative Visual Analog Scale (VAS), Athens Insomnia Scale (AIS), Numeric Rating Scale (NRS), Quality of Recovery-15 (QoR-15), Inpatient Cognitive Function Scale-10 (ICFS-10), remifentanil consumption), secondary (heart rate, mean arterial pressure, anesthesia duration, awakening time, and PACU stay);

Study Design: RCTs (randomized controlled trials).

Inclusion criteria comprised: (1) patients undergoing diverse laparoscopic procedures under general anesthesia; (2) perioperative intravenous esketamine; (3) Randomized controlled studies.

Exclusion criteria comprised: (1) non-RCTs including observational studies, case reports, conference abstracts, and reviews; (2) failure to specify esketamine administration protocols; (3) absence of perioperative outcome reporting (±3 days relative to surgery); (4) non-empirical publications (reviews, study protocols, consensus statements, announcements); (5) study participants younger than 18 years old (minors exhibit incomplete development of various physiological systems and significant inter-individual heterogeneity, which may lead to substantial variations in their responses to interventions and clinical outcomes).

### 2.3 Study selection

EndNote X9 automated reference curation with duplicate removal. Title/abstract screening initiated a two-stage appraisal process, with full-text evaluation of provisional candidates. Two researchers (W.S.H. and H.W.) independently screened the studies. They discussed and resolved any disagreements together. If they could not agree, another researcher (W.L.F.) made the final decision.

### 2.4 Data extraction

Two researchers systematically extracted data spanning bibliographic identifiers (lead author, publication year, geographic context), trial design elements, surgical typology, population characteristics (sex, age), and therapeutic regimens. The measured outcomes included VAS score, NRS score, AIS score, ICFS score, QoR-15 score, recovery time, heart rate, mean arterial pressure, Ramsay score, SAS score, extubation time, anesthesia duration, post-anesthesia care unit (PACU) time, remifentanil consumption, hospital stay duration, and plasma BDNF levels. In case of disagreement, a third researcher (W.L.F.) was invited to resolve the discrepancies.

### 2.5 Risk of bias

Two independent assessors executed bias assessment via the Cochrane RoB tool framework, methodically evaluating seven methodological domains: sequence generation, allocation concealment, blinding of participants and personnel, outcome assessment blinding, incomplete outcome data, selective outcome reporting, and other potential biases. Each item was assessed as “high risk,” “low risk,” or “unclear.” In cases of disagreement, a third researcher (W.L.F.) was invited to discuss and resolve the discrepancies.

### 2.6 Statistical analysis

The perioperative effects of esketamine in laparoscopic surgery were evaluated using descriptive statistics (mean ± SD) across 11 parameters: pain assessment (VAS, NRS, AIS), recovery indices (QoR-15, ICFS-10, awakening time), opioid requirements (remifentanil), procedural timelines (anesthesia duration, PACU stay), and hemodynamic measures (HR, MAP). Standardized mean differences (SMDs) with 95% confidence intervals (CIs) were computed for pooled outcomes. Heterogeneity criteria (χ^2^ P < 0.10; I^2^ > 50%) determined model selection, a random-effects model is utilized if heterogeneity is greater than 50%; conversely, a fixed-effects model is applied. Methodological robustness was validated through dual-platform analysis (RevMan 5.4/Stata 17), supplemented by sensitivity assessments via Stata-specific modules. When the included studies failed to directly report the mean ± SD, our primary approach was to reach out to the corresponding authors to request the raw data. In cases where the raw data could not be obtained, for studies presenting results as median (interquartile range, IQR), we applied the formula developed by Hozo (Hozo et al., 2005) et al. to estimate the mean and SD. For data with skewed distributions, logarithmic transformation was employed to standardize the effect sizes.

## 3 Results

### 3.1 Search results

**FIGURE 1:**
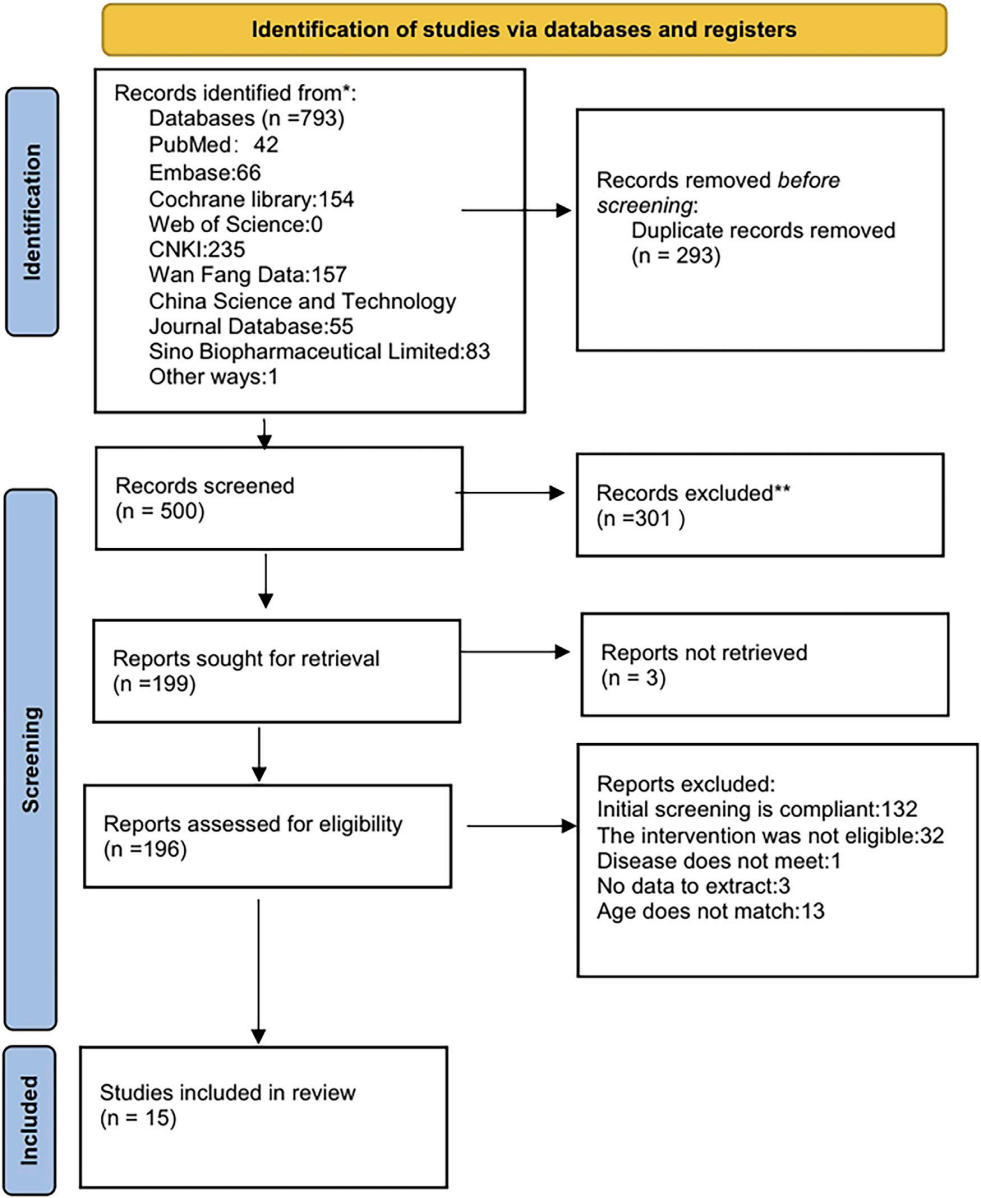
Flow diagram of study screening.

A systematic search of PubMed, Embase, Cochrane Library, Web of Science, China National Knowledge Infrastructure (CNKI), WanFang, VIP, and the China Biological Medicine Database (CBM) initially yielded 793 records. Automated deduplication via EndNote X9 eliminated 293 duplicates, leaving 500 unique citations for screening. Following title/abstract screening, 301 records were excluded, retaining 199 for full-text assessment. Full-text screening excluded 181 articles due to predefined criteria: exclusion of Chinese articles (n = 132), non-pharmacological interventions (n = 32), irrelevant pathologies (n = 1), insufficient outcome reporting (n = 3), and age criteria violations (n = 13). Fifteen studies were included in the final synthesis. The selection process was detailed in Figure 1.

### 3.2 Study characteristics

Fifteen RCTs enrolling 1,553 participants were analyzed, including 675 controls (placebo/standard anesthesia) and 878 intervention-group patients (esketamine). One trial featured a comparator subgroup of 104 patients receiving racemic ketamine. All trials were conducted in China (Zhao et al., 2024; Xu Y. et al., 2023; Dai et al., 2025; Hu et al., 2024; Huan et al., 2025; Jing et al., 2024; Lin et al., 2023; Lin et al., 2024; Liu et al., 2023; Qiu et al., 2022; Ren et al., 2024; Sun et al., 2023; Wang et al., 2020; Xu Z. et al., 2023; Zhang et al., 2023). The procedures encompassed laparoscopic approaches for gastrointestinal tumor resection (Xu Y. et al., 2023; Jing et al., 2024; Lin et al., 2024; Sun et al., 2023), bariatric surgery (Dai et al., 2025; Zhang et al., 2023), cholecystectomy (Zhao et al., 2024; Hu et al., 2024; Xu Z. et al., 2023), gynecological interventions (Huan et al., 2025; Lin et al., 2023; Liu et al., 2023; Wang et al., 2020), and renal procedures (Ren et al., 2024). Sample sizes varied between 19 and 105 subjects. Female-exclusive cohorts were observed in gynecological studies, whereas other trials enrolled mixed-sex populations. Esketamine was stratified into high- and low-dose regimens (Zhao et al., 2024; Dai et al., 2025; Huan et al., 2025; Lin et al., 2023; Ren et al., 2024; Wang et al., 2020), with all dosing delivered intravenously. Comprehensive methodological characteristics of the 15 studies are summarized in Table 1.

**TABLE 1:** Characteristics of the studies.

Study (Country)	Participants (the type of surgery)	Sample size, gender, age (melatonin/placebo)	Interventions (dose, route of administration, time)	Outcomes	Jadad score
Zhaojun Jing 2024 China	Laparoscopic gastrointestinal tumor surgery	Esketamine: 44, 30 male/14 female, 69.2 ± 6.22 No esketamine added: 43, 26 male/17 female, 71.47 ± 6.18 Data are mean ± SD	Esketamine: single dose 0.25 mg/kg and 0.1 mg/kg/h infusion intravenous infusion After induction of anesthesia and before surgical incision, Lasts until 30 min before the end of the procedure	VAS/AIS/QoR-15/Number of PCIA presses/IL-6	7
Jingyue Zhang 2023 China	Laparoscopic bariatric surgery	Esketamine: 35, 26 male/9 female, 30.17 ± 7.31 No esketamine added: 35, 24 male/11 female, 32.89 ± 8.16 Data are mean ± SD	Esketamine: infusion of 0.5 mg/kg/h, equivalent to 0.2 mL/kg/h intravenous infusion 20 min after tracheal intubation until the end of the procedure	NRS/Time to extubation/Length of hospital stay	7
Zhongling Xu 2023 China	Laparoscopic cholecystectomy	Esketamine: 27, 8 male/19 female, 56 (41, 67) No esketamine added: 27, 9 male/18 female, 55 (51, 61) Data are median and range	Esketamine: unclear intravenous infusion Before skin incision	VAS/Awakening time/Morphine dosage	7
Ying Xu 2023 China	radical laparoscopic colorectal cancer surgery	Esketamine: 45, 20 male/25 female, 57.0 (50.0, 59.0) No esketamine added: 43, 18 male/25 female, 56.0 (48.5, 59.0) Data are median and range	Esketamine: 0.25 mg/kg induction dose, continuous infusion of 0.12 mg/kg/h until surgical incision closure intravenous infusion Induction and until surgical incision closure	VAS/QoR-15/Time to extubation/Number of PCIA presses/Length of hospital stay/Remifentanil consumption/fluid administration/urine output/blood loss	5
Lei Sun 2023 China	radical laparoscopic colorectal cancer surgery	Esketamine: 32, 19 male/13 female, 60.16 ± 7.97 No esketamine added: 30, 17 male/13 female, 62.87 ± 8.37 Data are mean ± SD	Esketamine: 0.1 mg/kg·h intravenous infusion Pre-tracheal intubation until the end of the procedure	AIS/NRS/ICFS-10/Time to extubation/Length of hospital stay/Remifentanil consumption/Awakening time/blood loss/Ramsay	5
Tiantian Liu 2023 China	Laparoscopic gynecologic surgery	Esketamine: 20, all female, 46.25 ± 6.77 No esketamine added: 19, all female, 44.37 ± 9.74 Data are mean ± SD	Esketamine: 0.125 mg/kg intravenous infusion 30 min after the start of surgery	Remifentanil consumption/anesthesia time/SAS	4
Di Qiu 2022 China	Laparoscopic gynecologic surgery	Esketamine: 20, all female, 43 (32, 49) No esketamine added: 19, all female, 45 (35, 49) Data are median and range	Esketamine: 0.3 mg/kg/h intravenous infusion After anesthesia until the end of surgery	VAS/AIS/NRS/Remifentanil consumption/PACU time	7
Jiabao Dai 2024 China	Laparoscopic bariatric surgery	Postoperative esketamine group: 37, 11 male/26 female, 31.2 ± 6.7 Preoperative esketamine group: 40, 6 male/34 female, 32.2 ± 7.1 No esketamine added:36,7 male/29 female, 31.8 ± 7.9 Data are mean ± SD	Esketamine: 0.2 mg/kg intravenous infusion Preoperative and 2 h postoperative	VAS/QoR-15/Time to extubation/Number of PCIA presses/Length of hospital stay /BDNF	5
Lu Zhao 2024 China	Laparoscopic cholecystectomy	High-dose esketamine group: 30, 15 male/15 female, 39.5 ± 10.6 Low-dose esketamine group: 30, 16 male/14 female, 40.3 ± 10.3 No esketamine added: 30, 14 male/16 female, 39.3 ± 11.2 Data are mean ± SD	High-dose esketamine group: 0.5 mg/kg, 4 μg/kg·min Low-dose esketamine group: 0.5 mg/kg, 2 μg/kg·min intravenous infusion Subsequent continuous infusion prior to initial skin incision	IL-6/HR/MAP/Remifentanil consumption/Awakening time/Surgical time /anesthesia time	5
Jie Wang 2020 China	laparoscopic total hysterectomy	High-dose esketamine group: 104, all female, 48.53 ± 10.0 Low-dose esketamine group: 104, all female, 48.11 ± 10.38 Racemic ketamine group: 104, all female, 47.07 ± 10.08 No esketamine added: 105, all female, 46.27 ± 10.83 Data are mean ± SD	High-dose esketamine group: 0.5 mg/kg Low-dose esketamine group: 0.25 mg/kg intravenous infusion Patient after 1 h of analgesia	Length of hospital stay/blood loss/Surgical time/BDNF	6
Liyuan Ren 2024 China	Laparoscopic renal surgery	High-dose esketamine group: 40, 18 male/22 female, 51.0 (40.0, 56.0) Low-dose esketamine group: 40, 21 male/19 female, 54.0 (42.0, 61.0) No esketamine added: 40, 23 male/17 female, 52.0 (45.0, 59.0) Data are median and range	High-dose esketamine group: 0.25 mg/kg/h Low-dose esketamine group: 0.125 mg/kg/h intravenous infusion Under stabilized anesthesia and surgery, the patient is started on a continuous infusion of	QoR-15/NRS/Time to extubation/Remifentanil consumption/fluid administration/urine output/blood loss/Surgical time/anesthesia time/SAS/PACU time	5
Li Lin 2023 China	Laparoscopic resection of benign ovarian tumors	High-dose esketamine group: 35, all female, 40.21 ± 3.22 Low-dose esketamine group: 37, all female, 40.19 ± 3.20 No esketamine added: 38, all female, 40.14 ± 3.16 Data are mean ± SD	High-dose esketamine group: 0.8 mg/kg Low-dose esketamine group: 0.6 mg/kg intravenous infusion At induction of anesthesia	VAS/Time to extubation/HR/MAP/Awakening time/Morphine dosage/Surgical time	4
Chen Huan 2025 China	Laparoscopic gynecologic surgery	High-dose esketamine group: 40, all female, 49 ± 7 Low-dose esketamine group: 39, all female, 49 ± 8 No esketamine added: 41, all female, 48 ± 9 Data are mean ± SD	High-dose esketamine group: 0.50 mg/kg, 0.40 mg/kg/h Low-dose esketamine group: 0.25 mg/kg, 0.20 mg/kg/h intravenous infusion Prior to traumatic incision; throughout	NRS	7
Xinru Lin 2024 China	laparoscopic gastrectomy for gastric cancer	Esketamine: 62,45 male/17 female, 64.30 ± 10.40 No esketamine added: 62, 43 male/19 female, 66.81 ± 11.27 Data are mean ± SD	Esketamine: 0.5 mg/kg; PCIA 1 mg/kg intravenous infusion During induction of anesthesia and PCIA	VAS/Length of hospital stay/ICFS-10	
Fanyan Hu 2021 China	laparoscopic cholecystectomy	Esketamine: 35, 15 male/20 female, 45.17 ± 10.26 No esketamine added: 35, 16 male/19 female, 46.34 ± 8.91 Data are mean ± SD	Esketamine: 0.4 mg/kg; 0.1 mg/kg/h intravenous infusion During induction and maintenance of anesthesia	VAS/HR/Awakening time/Ramsay/PACU time	6

Abbreviations: SD, standard deviation; IQR, interquartile range.

### 3.3 Risk of bias

**FIGURE 2:**
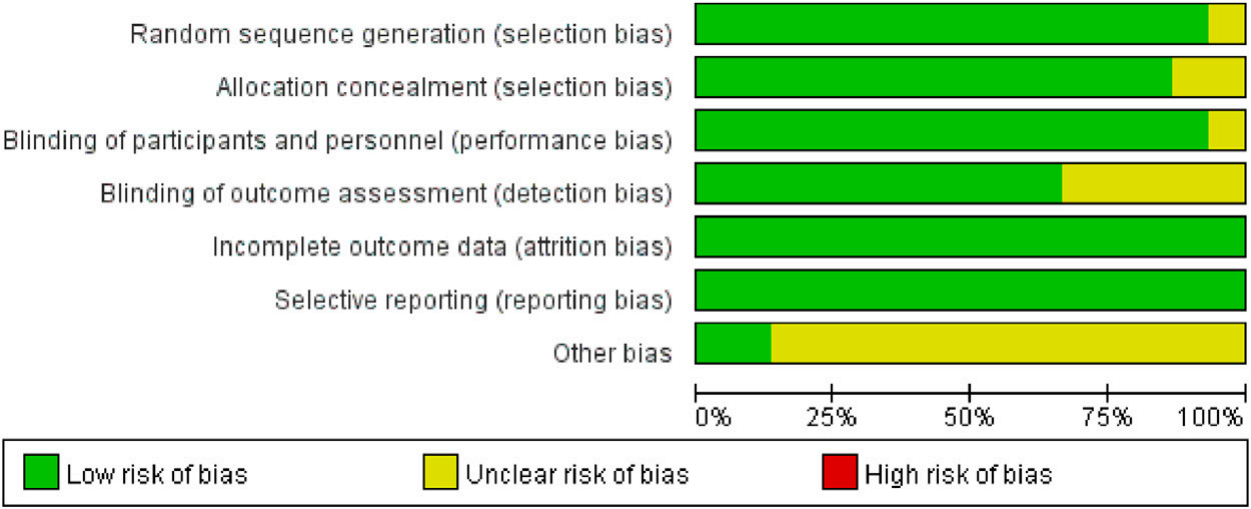
Risk of bias graph.

**FIGURE 3:**
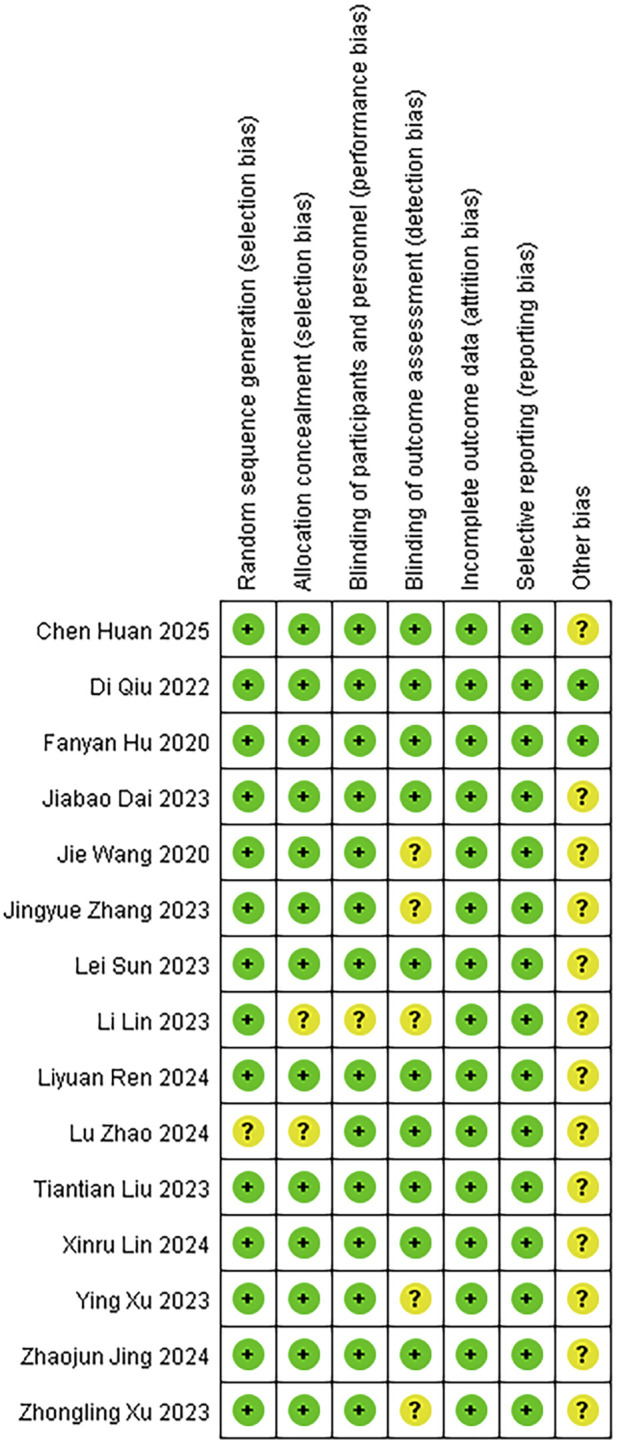
Risk of bias summary.

We assessed the risk of bias for 15 studies using RevMan 5.4. The overall risk of bias is illustrated in Figure 2, while the detailed risk of bias for each individual study is presented in Figure 3. One study (Zhao et al., 2024) exhibited an unclear risk of bias owing to inadequate details on random sequence generation, whereas the other 14 studies showed low risk in this domain. Two studies (Zhao et al., 2024; Lin et al., 2023) exhibited an uncertain risk of selection bias owing to insufficient allocation concealment, whereas the other 13 studies displayed a low risk in this domain. One study (Lin et al., 2023) was assessed as having an unclear risk of selection bias regarding investigator/participant blinding, whereas the remaining 13 studies showed low risk in this methodological component. The remaining 13 trials demonstrated robust blinding integrity with low risk. Five studies (Xu Y. et al., 2023; Lin et al., 2023; Wang et al., 2020; Xu Z. et al., 2023; Zhang et al., 2023) exhibited an unclear risk of bias in outcome assessment owing to insufficient documentation of assessors’ awareness of interventions, whereas the remaining 10 studies demonstrated low risk in this methodological domain. All included studies demonstrated minimal outcome reporting bias. Methodological rigor in non-core bias domains was confirmed for trials (Hu et al., 2024; Qiu et al., 2022) following structured appraisal of full-text documentation. Six trials (Zhao et al., 2024; Jing et al., 2024; Lin et al., 2023; Liu et al., 2023; Sun et al., 2023; Xu Z. et al., 2023) utilized samples of moderate scale without prior power calculations, resulting in compromised statistical power for secondary endpoints and subsequent designation of methodological uncertainty in ancillary bias domains. Zhang’s trial (Zhang et al., 2023) documented intraoperative hemodynamic fluctuations and artificial intelligence (AI)-associated analytical confounders, yielding designation of methodological uncertainties in ancillary bias domains. Xu’s trial (Xu Y. et al., 2023) employed limited sample sizes inadequate for secondary outcome validation, subsequently categorized as underpowered with ancillary bias potential. Studies (Huan et al., 2025; Lin et al., 2024) lacked multicenter validation frameworks, classified as methodological constraints in generalizability domains. Dai’s protocol (Dai et al., 2025) exhibited structural vulnerabilities where trial design inadvertently prompted participant anticipation of therapeutic interventions, warranting designation of directional confounding risks. Ren’s investigation (Ren et al., 2024) lacked generalizability assessments across anesthetic agents (e.g., propofol/desflurane), yielding designation of methodological uncertainties in ancillary bias domains. Wang’s trial (Wang et al., 2020) exhibited insufficient documentation of non-core confounding variables, classified as potential constraints in bias control frameworks. In this study, given the exclusive inclusion of RCTs, a comprehensive quality assessment was conducted for all enrolled trials utilizing the Jadad rating scale (also referred to as the Oxford Quality Scoring System). This validated instrument evaluates the methodological rigor of RCTs by examining three critical domains: randomization procedures, blinding implementation, and handling of withdrawals and dropouts. With a maximum achievable score of 5 points, the Jadad scale has gained widespread recognition in methodological research for its simplicity, practicality, and reliability in assessing potential biases in clinical trials (Zhao et al., 2024; Xu Y. et al., 2023; Dai et al., 2025; Hu et al., 2024; Huan et al., 2025; Jing et al., 2024; Lin et al., 2023; Lin et al., 2024; Liu et al., 2023; Qiu et al., 2022; Ren et al., 2024; Sun et al., 2023; Wang et al., 2020; Xu Z. et al., 2023; Zhang et al., 2023). The systematic application of this tool ensured a robust evaluation of the included studies’ quality.

### 3.4 Meta-analysis

#### 3.4.1 Postoperative visual analog scale (VAS) scores

**FIGURE 4:**
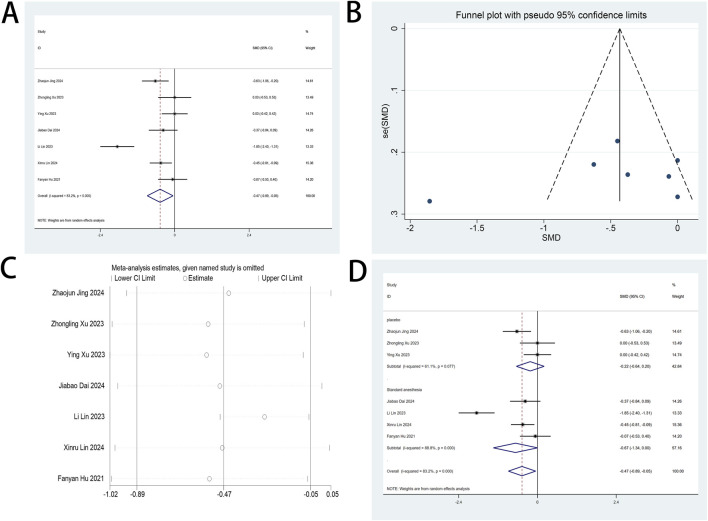
**(A)** Forest plot to assess the effect of esketamine on VAS scores; **(B)** Funnel plot to assess the effect of esketamine on VAS scores; **(C)** Sensitivity analysis to assess the effect of esketamine on VAS scores; **(D)** Forest plot for subgroup analysis of VAS scores.

This systematic review prioritized VAS metrics as its principal efficacy endpoint. Seven randomized investigations (Xu Y. et al., 2023; Dai et al., 2025; Hu et al., 2024; Jing et al., 2024; Lin et al., 2023; Lin et al., 2024; Xu Z. et al., 2023) quantified esketamine’s analgesic efficacy through standardized VAS measurements. Pooled analysis revealed clinically meaningful VAS reductions on postoperative day 1 (SMD: −0.47; 95% CI [−0.89, −0.05], P = 0.027), substantiating esketamine’s role in pain mitigation (Figure 4A). High heterogeneity was observed (I^2^ = 83.2%, P < 0.001; Figure 4A). Funnel plot asymmetry demonstrated detectable publication bias (Figure 4B). Sensitivity analyses demonstrated invariant pooled estimates across sequential study exclusions, confirming analytical stability (Figure 4C). Subgroup analyses were conducted based on the composition of the control group. However, the results of these analyses did not reach statistical significance, indicating no discernible differences across the subgroups examined (Figure 4D). Postoperative VAS metrics at POD 2-3 and cough-associated measurements revealed no statistically discernible differences versus controls (Supplementary Figure S1-S6).

#### 3.4.2 Postoperative numeric rating scale (NRS) scores

**FIGURE 5:**
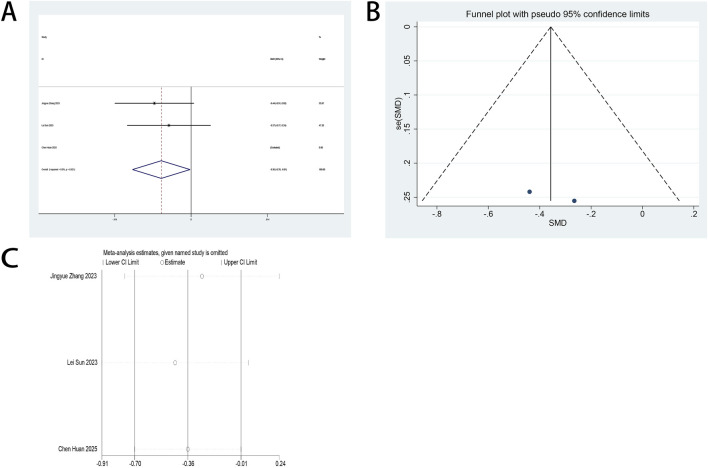
**(A)** Forest plot to assess the effect of esketamine on NRS scores; **(B)** Funnel plot to assess the effect of esketamine on NRS scores; **(C)** Sensitivity analysis to assess the effect of esketamine on NRS scores.

The NRS serves as a primary outcome measure in this systematic review, providing quantifiable assessments of postoperative pain intensity. Five RCTs (Huan et al., 2025; Jing et al., 2024; Qiu et al., 2022; Ren et al., 2024; Sun et al., 2023) quantified esketamine’s therapeutic impact on NRS metrics among minimally invasive surgery cohorts. Pooled analysis of the five trials demonstrated statistically significant reductions in NRS metrics at postoperative day 2 following esketamine administration (SMD: −0.36; 95% CI [−0.70, −0.01], P = 0.042; Figure 5A). No heterogeneity was confirmed (I^2^ = 0%, P = 0.621; Figure 5A), while the funnel plot revealed detectable publication bias (Figure 5B). Sequential exclusion sensitivity analysis validated methodological stability (Figure 5C). Comparative analysis of POD 1, 3, and 7 measurements showed clinically comparable NRS outcomes versus controls (Supplementary Figure S7-S11).

#### 3.4.3 Postoperative athens insomnia scale (AIS) scores

**FIGURE 6:**
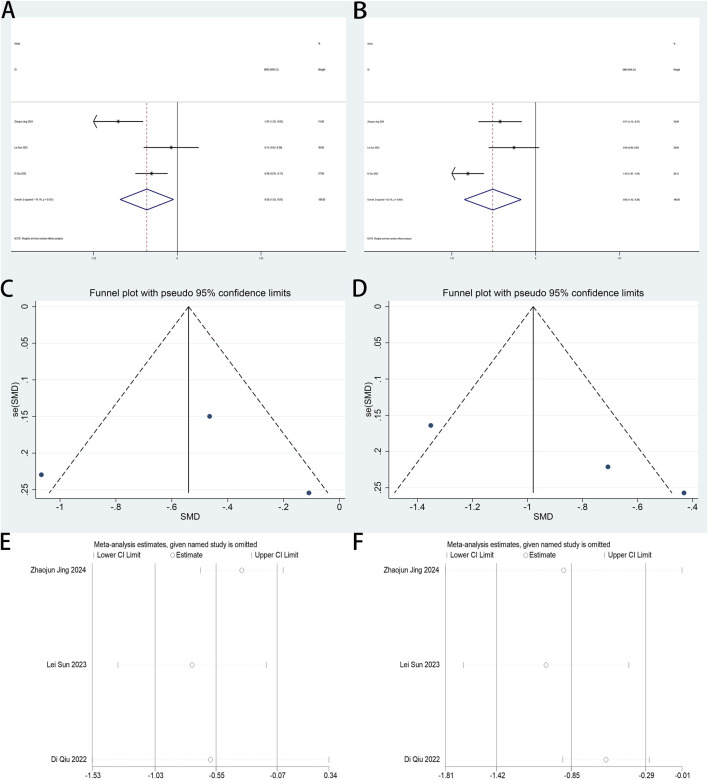
**(A)** Forest plot to assess the effect of esketamine on AIS scores on the first postoperative day; **(B)** Forest plot to assess the effect of esketamine on AIS scores on the third postoperative day; **(C)** Funnel plot to assess the effect of esketamine on AIS scores on the first postoperative day; **(D)** Funnel plot to assess the effect of esketamine on AIS scores on the third postoperative day; **(E)** Sensitivity analysis to assess the effect of esketamine on AIS scores on the first postoperative day; **(F)** Sensitivity analysis to assess the effect of esketamine on AIS scores on the third postoperative day.

The AIS serves as a primary outcome measure in this systematic review. Three RCTs (Jing et al., 2024; Qiu et al., 2022; Sun et al., 2023) quantified esketamine’s therapeutic impact on postoperative sleep quality among minimally invasive surgical cohorts through standardized AIS assessments. Pooled analysis demonstrated statistically significant enhancements in postoperative sleep quality following esketamine administration, with standardized mean differences (SMD: −0.55; 95% CI [−1.03, −0.07], P = 0.026) at POD 1 and (SMD: −0.85; 95% CI [−1.42, −0.29], P = 0.003) at POD 3 (Figures 6A,B). High heterogeneity was detected across both timepoints (POD 1: I^2^ = 76.1%, P = 0.015; POD 3: I^2^ = 82.1%, P = 0.004; Figures 6A,B). Funnel plot asymmetries revealed detectable publication bias (Figures 6C,D). Analytical stability was validated through sequential exclusion sensitivity testing, maintaining invariant effect estimates (Figures 6E,F).

#### 3.4.4 Postoperative quality of recovery (QoR-15)

**FIGURE 7:**
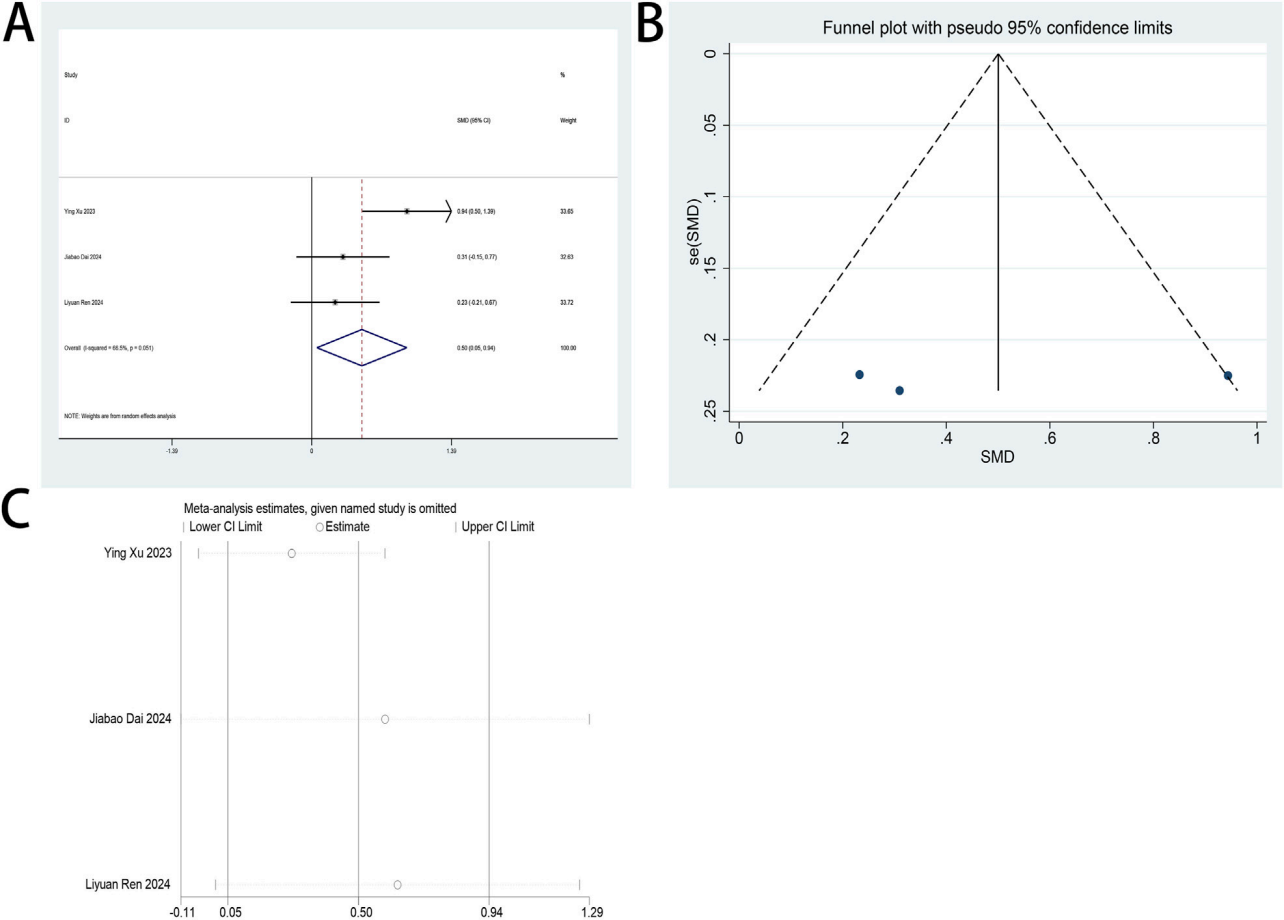
**(A)** Forest plot to assess the effect of esketamine on QoR-15 scores on the first postoperative day; **(B)** Funnel plot to assess the effect of esketamine on QoR-15 scores on the first postoperative day; **(C)** Sensitivity analysis to assess the effect of esketamine on QoR-15 scores on the first postoperative day.

The QoR-15 score constitutes the primary endpoint in this review. Four studies (Xu Y. et al., 2023; Dai et al., 2025; Jing et al., 2024; Ren et al., 2024) assessed esketamine’s effect on postoperative recovery quality in laparoscopic surgical cohorts using this validated metric. The combined analysis of four trials demonstrated esketamine’s enhancement of postoperative recovery quality by day 1 (SMD: −0.50; 95% CI [0.05, 0.94]; P = 0.029; Figure 7A). High heterogeneity was identified across studies (P = 0.051, I^2^ = 66.5%; Figure 7A). Funnel plot analysis revealed potential publication bias (Figure 7B). Sensitivity analysis demonstrated consistent meta-analysis outcomes across all trial exclusions, confirming result robustness (Figure 7C). Esketamine exhibited no statistically significant impact on postoperative day 2 QoR-15 scores versus controls (Supplementary Figure S12).

#### 3.4.5 Perioperative fatigue

**FIGURE 8:**
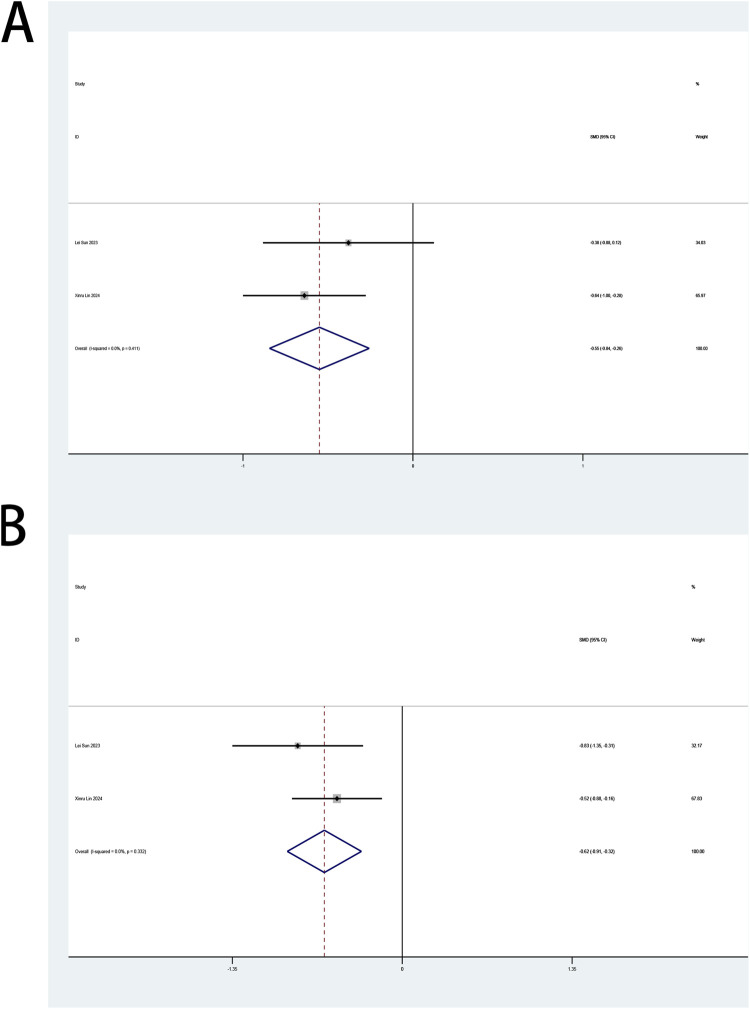
**(A)** Forest plot to assess the effect of esketamine on ICFS-10 scores on the first postoperative day; **(B)** Forest plot to assess the effect of esketamine on ICFS-10 scores on the third postoperative day.

The perioperative ICFS-10 serves as a primary endpoint in this review. Two trials (Lin et al., 2024; Sun et al., 2023) examined esketamine’s impact on fatigue management in patients undergoing laparoscopic surgery. A pooled analysis of both trials, stratified by postoperative intervals (days 1, 3, 7), demonstrated esketamine’s therapeutic efficacy in mitigating early postoperative fatigue (days 1–3), with significant reductions in ICFS-10 scores. Esketamine demonstrated sustained analgesic efficacy across postoperative days 1 (SMD: −0.55; 95% CI [−0.84, −0.26]; P < 0.001; Figure 8A) and 3 (SMD: −0.62; 95% CI [−0.91, −0.32]; P < 0.001; Figure 8B), with SMDs exceeding predefined clinical significance thresholds. No heterogeneity between studies was shown for both day 1 (P = 0.411, I^2^ = 0.0%; Figure 8A) and day 3 (P = 0.332, I^2^ = 0.0%; Figure 8B). Compared with controls, esketamine demonstrated no statistically significant impact on fatigue scores at postoperative day 7 (Supplementary Figure S13).

#### 3.4.6 Intraoperative remifentanil consumption

Intraoperative remifentanil utilization served as a secondary endpoint in this systematic review. Six trials (Zhao et al., 2024; Xu Y. et al., 2023; Liu et al., 2023; Qiu et al., 2022; Ren et al., 2024; Sun et al., 2023) assessed esketamine’s opioid-sparing effects during laparoscopic procedures. A pooled analysis of six trials (Zhao et al., 2024; Xu Y. et al., 2023; Liu et al., 2023; Qiu et al., 2022; Ren et al., 2024; Sun et al., 2023) stratified remifentanil dosing parameters through a standardized analytical framework: four studies (Zhao et al., 2024; Xu Y. et al., 2023; Liu et al., 2023; Sun et al., 2023) by cumulative infusion totals and two (Qiu et al., 2022; Ren et al., 2024) by time-adjusted infusion rates, addressing heterogeneous measurement units across datasets.

**FIGURE 9:**
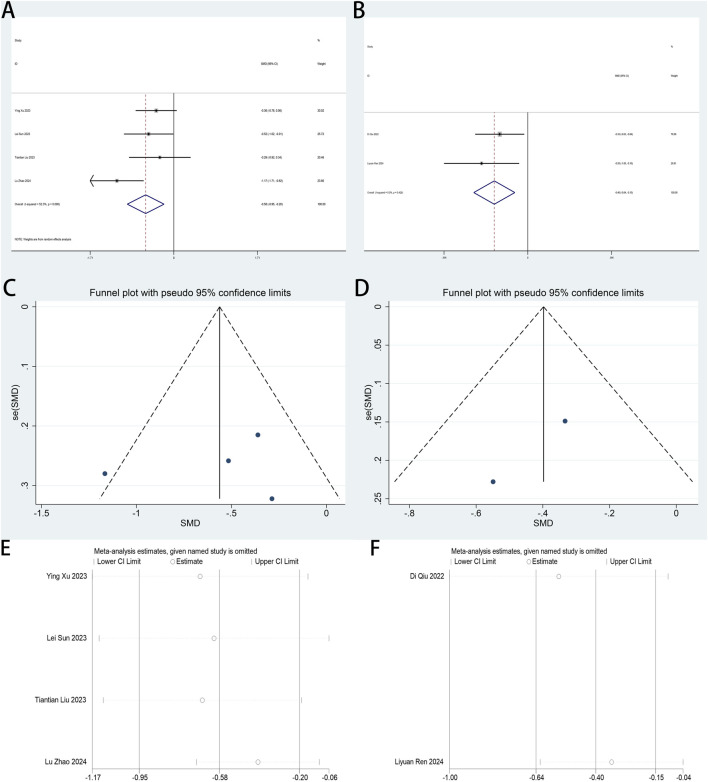
**(A)** Forest plot to assess the effect of esketamine on total intraoperative remifentanil consumption; 9 **(B)** Forest plot to assess the effect of esketamine on intraoperative remifentanil infusion per unit of time; 9 **(C)** Funnel plot to assess the effect of esketamine on total intraoperative remifentanil infusions; **(D)** Funnel plot to assess the effect of esketamine on intraoperative remifentanil infusion per unit of time; **(E)** Sensitivity analysis to assess the effect of esketamine on total intraoperative remifentanil infusions; **(F)** Sensitivity analysis to assess the effect of esketamine on intraoperative remifentanil infusion per unit of time.

Meta-analysis demonstrated esketamine’s significant opioid-sparing efficacy in laparoscopic procedures, with SMDs (SMD: −0.58; 95% CI [−0.95, −0.20]; P < 0.01; Figure 9A) revealing reduced intraoperative remifentanil requirements. High heterogeneity was observed (I^2^ = 52.3%, P = 0.098). Esketamine administration significantly attenuated time-normalized remifentanil infusion rates (SMD: −0.40; 95% CI [-0.64, −0.15]; P < 0.01; Figure 9B), with negligible interstudy heterogeneity (I^2^ = 0.0%, P = 0.428). Funnel plot analysis revealed potential publication bias across both dosing metrics (Figures 9C,D). Sensitivity analysis confirmed methodological stability across the six included trials, with sequential exclusion producing no significant alterations in meta-analytic outcomes (Figures 9E,F), indicating result robustness.

#### 3.4.7 Perioperative plasma brain-derived neurotrophic factor (BDNF) levels

**FIGURE 10:**
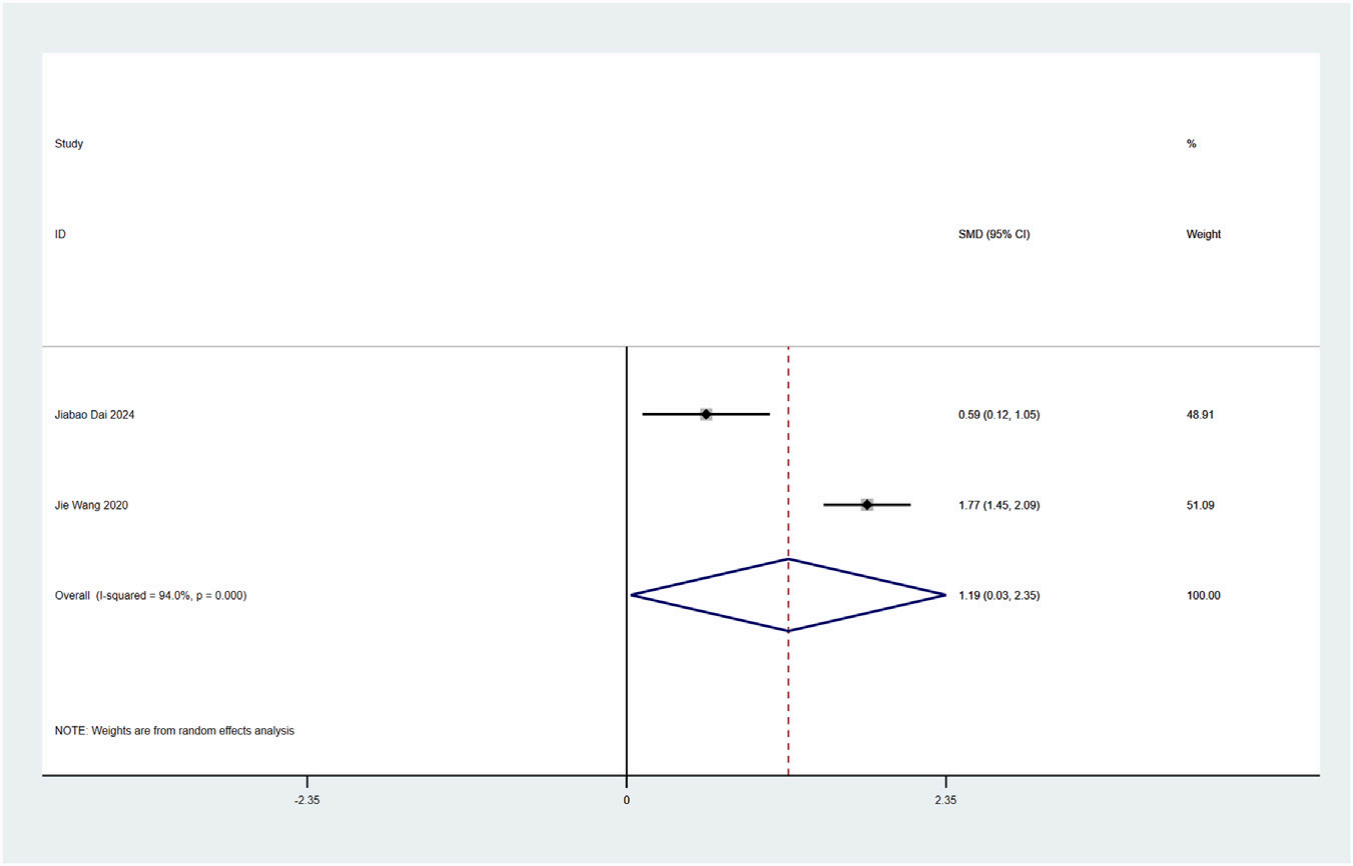
Forest plot to assess the effect of esketamine on postoperative plasma BDNF factor concentrations.

Perioperative plasma BDNF concentration served as a primary endpoint in this systematic review. Two RCTs (Dai et al., 2025; Wang et al., 2020) examined esketamine’s neuroplastic modulation through postoperative BDNF quantification in laparoscopic cohorts. Meta-analytic synthesis demonstrated significant BDNF elevation on postoperative day 2 (SMD: 1.19; 95% CI [0.03, 2.35]; P = 0.04; Figure 10).

High heterogeneity was observed (I^2^ = 94%, P < 0.001; Figure 10). Funnel plot analysis suggested potential publication bias, while comparative analysis revealed no statistically significant perioperative plasma BDNF level alterations following esketamine administration versus controls, particularly on postoperative day 1 (Supplementary Figure S14).

#### 3.4.8 Perioperative heart rate, mean arterial pressure, urine output, fluid intake, blood loss, awakening time, ramsay score, SAS score, PCIA button presses, extubation time, PACU time, anesthesia time, morphine consumption, length of hospital stay, and serum IL-6 levels

Secondary endpoints comprised four clinical domains: (1) hemodynamic parameters (heart rate, mean arterial pressure), (2) perioperative management metrics (fluid balance [urine output, fluid intake], blood loss), (3) recovery indices (emergence time, extubation time, PACU duration, anesthesia time), and (4) pharmacodynamic outcomes (Ramsay/SAS sedation scores, PCIA demand frequency, morphine equivalents utilization). Additional exploratory measures included postoperative interleukin-6 concentrations and hospitalization length (Lin et al., 2023; Ren et al., 2024).

Three RCTs (Zhao et al., 2024; Hu et al., 2024; Lin et al., 2023) investigated esketamine’s perioperative cardiophysiological impacts, with meta-analytic synthesis demonstrating no significant alterations in laparoscopic patients’ heart rates (SMD: −0.18; 95% CI [−0.46, 0.10]; P = 0.20) under no heterogeneity (I^2^ = 0.0%, P = 0.46) (Supplementary Figure S15).

Two RCTs (Zhao et al., 2024; Lin et al., 2023) examined esketamine’s hemodynamic effects through postoperative mean arterial pressure (MAP) monitoring in laparoscopic cohorts. Meta-analytic integration revealed non-significant MAP alterations following esketamine administration (SMD: −0.30; 95% CI [−0.86, 0.27); P = 0.30), with high heterogeneity observed across studies (I^2^ = 63.2%, P = 0.10) (Supplementary Figure S16).

Two RCTs (Xu Y. et al., 2023; Ren et al., 2024) assessing esketamine’s renal effects during laparoscopy revealed meta-analytically significant increases in postoperative urine output (SMD: 0.33; 95% CI [0.03, 0.64]; P = 0.03) under no heterogeneity (I^2^ = 43.7%, P = 0.18). Funnel plot asymmetry suggested potential publication bias among included studies (Supplementary Figure S17, S18).

Two RCTs (Xu Y. et al., 2023; Ren et al., 2024) investigating esketamine’s perioperative fluid management impacts in laparoscopic surgery demonstrated meta-analytically significant increases in intraoperative fluid administration volumes (SMD: 0.55; 95% CI [0.24, 0.86]; P < 0.001) with no heterogeneity (I^2^ = 0.0%, P = 0.41). Funnel plot asymmetry suggested potential publication bias among included studies (Supplementary Figure S17, S18).

Four RCTs (Xu Y. et al., 2023; Ren et al., 2024; Sun et al., 2023; Wang et al., 2020) investigating esketamine’s intraoperative hemostatic efficacy in laparoscopic surgery demonstrated meta-analytically non-significant blood loss outcomes (SMD: 0.04; 95% CI [-0.15, 0.23]; P = 0.69) under no heterogeneity (I^2^ = 0.0%, P = 0.98) (Supplementary Figure S21-S23).

Two RCTs (Zhao et al., 2024; Jing et al., 2024) investigating esketamine’s immunomodulatory effects during laparoscopy demonstrated meta-analytically non-significant alterations in postoperative serum interleukin-6 (IL-6) concentrations (SMD: −0.16; 95% CI [-0.48, 0.16]; P = 0.33) with no heterogeneity (I^2^ = 0.0%, P = 0.50) (Supplementary Figure S24).

Five RCTs (Zhao et al., 2024; Hu et al., 2024; Lin et al., 2023; Sun et al., 2023; Xu Z. et al., 2023) investigating esketamine’s pharmacodynamic impact on perioperative recovery in laparoscopy revealed meta-analytically non-significant alterations in postoperative emergence time (SMD: −0.38; 95% CI [−0.93, 0.18]; P = 0.19) despite high heterogeneity (I^2^ = 83.6%, P < 0.001) (Supplementary Figures S25-S27). Notably, the studies by Xu (Xu Z. et al., 2023) and Zhao (Zhao et al., 2024) employed varying definitions, which stood in contrast to the other three articles (Hu et al., 2024; Lin et al., 2023; Sun et al., 2023), where no explicit definitions were provided. This lack of uniformity in defining key concepts across the studies likely contributed to the high heterogeneity observed.

Two RCTs (Liu et al., 2023; Ren et al., 2024) investigating the anxiolytic potential of perioperative esketamine in laparoscopy, quantified through the Self-Rating Anxiety Scale (SAS), demonstrated meta-analytically non-significant psychiatric outcomes (SMD: 0.00; 95% CI [-0.36, 0.36]; P = 1.00) with no heterogeneity (I^2^ = 0.0%, P = 1.00) and absent publication bias (Supplementary Figure S28).

Two RCTs (Hu et al., 2024; Sun et al., 2023) investigating the sedative efficacy of perioperative esketamine in laparoscopy, assessed via the Ramsay Sedation Scale (RSS), meta-analytically demonstrated non-significant postoperative recovery outcomes (SMD: 0.76; 95% CI [-0.74, 2.26]; P < 0.001) despite high heterogeneity (I^2^ = 94.1%, P < 0.001) (Supplementary Figure S29).

Three RCTs (Xu Y. et al., 2023; Dai et al., 2025; Jing et al., 2024) investigating the analgesic efficacy of perioperative esketamine in laparoscopy meta-analytically demonstrated non-significant reductions in postoperative patient-controlled analgesia (PCA) utilization (SMD: −0.51; 95% CI [-1.51, 0.49]; P = 0.32) despite high between-study heterogeneity (I^2^ = 93.2%, P < 0.001) (Supplementary Figure S30).

Six RCTs (Xu Y. et al., 2023; Dai et al., 2025; Lin et al., 2023; Ren et al., 2024; Sun et al., 2023; Zhang et al., 2023) investigating the pharmacological modulation of perioperative extubation dynamics in laparoscopy meta-analytically demonstrated non-significant alterations in tracheal decannulation latency (SMD: −0.02; 95% CI [-0.34, 0.30]; P = 0.89) despite high heterogeneity (I^2^ = 66.0%, P = 0.012) (Supplementary Figure S31-S33).

Three RCTs (Hu et al., 2024; Qiu et al., 2022; Ren et al., 2024) investigating the pharmacological impact of perioperative esketamine on post-anesthesia care unit (PACU) residency duration meta-analytically demonstrated non-significant alterations in recovery facility occupancy intervals (SMD: 0.28; 95% CI [-0.37, 0.93]; P = 0.40) despite high heterogeneity (I^2^ = 87.5%, P < 0.001) (Supplementary Figure S34).

Two RCTs (Lin et al., 2023; Xu Z. et al., 2023) investigating the opioid-sparing properties of perioperative esketamine in laparoscopy meta-analytically demonstrated non-significant alterations in postoperative opioid requirements (SMD: 0.01; 95% CI [-0.34, 0.36]; P = 0.96) with no heterogeneity (I^2^ = 0.0%, P = 0.89) (Supplementary Figure S35).

Three RCTs (Zhao et al., 2024; Liu et al., 2023; Ren et al., 2024) investigating the pharmacological modulation of esketamine on procedural sedation duration in laparoscopic surgery meta-analytically demonstrated non-significant alterations in total anesthesia exposure (SMD: −0.10; 95% CI [−0.39, 0.19]; P = 0.51) with no heterogeneity (I^2^ = 0.0%, P = 0.41) (Supplementary Figure S36).

Five RCTs (Zhao et al., 2024; Lin et al., 2023; Ren et al., 2024; Sun et al., 2023; Wang et al., 2020) examined esketamine’s impact on operative duration in laparoscopic procedures. Meta-analysis revealed no significant effect on surgical time (SMD: 0.08; 95% CI [−0.10, 0.26]; P = 0.37), with no significant heterogeneity observed (I^2^ = 0.0%, P = 0.98) (Supplementary Figures S37-S39).

Six RCTs (Xu Y. et al., 2023; Dai et al., 2025; Lin et al., 2024; Sun et al., 2023; Wang et al., 2020; Zhang et al., 2023) assessed esketamine’s impact on postoperative hospitalization duration. Meta-analysis demonstrated no significant effect on this outcome (SMD: −0.10; 95% CI [−0.26, 0.05; P = 0.20), with no heterogeneity observed (I^2^ = 28.9%, P = 0.22) (Supplementary Figures S40-S42).

## 4 Discussion

### 4.1 Interpretation of results

#### 4.1.1 Primary outcome measures

This study demonstrates that esketamine significantly reduces postoperative pain in laparoscopic surgery patients, as shown by marked decreases in VAS and NRS scores. These findings align with evidence from recent meta-analyses (Xie et al., 2023; Niu et al., 2024; Wen et al., 2025; Wang X. et al., 2021). Esketamine reduces intraoperative remifentanil consumption, thereby decreasing total opioid requirements and associated risks of respiratory depression and postoperative nausea/vomiting. Compared with opioid medications, ketamine exerts no significant inhibitory effect on respiration. From the perspective of clinical practice, the mechanism of action underlying the continuous intravenous infusion of ketamine merits special attention. Specifically, it selectively blocks N-methyl-D-aspartate (NMDA) receptors, which in turn effectively suppresses the overactivation of the ascending nociceptive pathway and reduces the release of pain-inducing substances (e.g., bradykinin and substance P) at the spinal cord level. Consequently, ketamine exhibits a prominent “anti-hyperalgesic” effect and is capable of preventing and reversing central sensitization. In clinical settings, this dosing regimen is particularly well-suited for patients who are anticipated to experience severe postoperative pain, have a history of chronic pain, are at risk of developing hyperalgesia, or require long-term opioid administration (Zhang et al., 2024; Wang H. et al., 2021; Viisanen et al., 2020). Furthermore, it enhances patient-reported recovery quality, consistent with Hung et al. (Hung et al., 2024), who demonstrated esketamine’s efficacy in mitigating both acute and chronic postoperative pain. Esketamine administration was observed to modestly elevate plasma BDNF concentrations in postoperative laparoscopic patients, a phenomenon potentially linked to its perioperative antidepressant properties shared with ketamine. This biochemical association aligns with contemporary mechanistic studies (Medeiros et al., 2022; Reif et al., 2023). However, owing to the limited inclusion of literature data in this meta-analysis, no evidence was found to support that ketamine can alleviate perioperative depressive symptoms in patients undergoing laparoscopic surgery. This study identified esketamine’s capacity to alleviate postoperative fatigue in laparoscopic patients, evidenced by reduced ICFS-10 scores. Notably, this finding lacks corroboration in existing meta-analyses. In addition, there is currently a lack of multiple randomized controlled trials (RCTs) to support the claim that ketamine can significantly reduce the incidence of postoperative fatigue in patients undergoing laparoscopic surgery. Consequently, large-scale clinical trials and extended postoperative follow-up are required to validate this potential effect. Moreover, esketamine concurrently enhanced postoperative sleep quality and mitigated insomnia incidence in this cohort, aligning with outcomes reported in recent trials (Niu et al., 2024; Kwaśny et al., 2023). The current evaluation framework utilizing the AIS failed to establish esketamine as the optimal intervention for enhancing postoperative sleep quality in laparoscopic patients, necessitating further mechanistic and comparative clinical investigations.

#### 4.1.2 Secondary outcome measures

This systematic review evaluates several secondary outcomes, including perioperative heart rate, mean arterial pressure, urine output, fluid intake, awakening time, blood loss, Ramsay and SAS scores, PCIA press counts, extubation time, PACU stay, anesthesia duration, morphine consumption, hospital length of stay, and serum IL-6 levels. Heart rate, mean arterial pressure, blood loss, anesthesia duration, hospital stay, awakening time, PACU stay, PCIA press counts, and morphine consumption are subject to multiple confounding factors, such as surgical type, environmental conditions, and individual variability. Despite esketamine administration, these influences persist, suggesting esketamine exerts no direct effect on these parameters. Our findings indicate that esketamine increases intraoperative fluid intake and urine output in laparoscopic surgery patients, likely due to their proportional relationship, though the underlying mechanism remains unclear. Regarding sedation, esketamine had no impact on Ramsay and SAS scores in laparoscopic surgery patients, aligning with Yao’s study (Yao et al., 2024) on postoperative analgesia-related sedation. However, Xu LL. et al. (2023) reported enhanced sedation with esketamine in cesarean section patients, contradicting our findings. Esketamine also showed no significant effect on postoperative emotional state, as assessed by SAS scores. This contrasts with findings from Qu et al. (2025) and Li et al. (2024), who observed different outcomes in hip and thyroidectomy patients, likely due to variations in surgical procedures, patient populations, or perioperative factors. Our results differ from some existing RCTs, potentially due to data extraction methods that may introduce inaccuracies. Given the limited number of included studies, further research is needed to confirm these findings.

### 4.2 Strengths of the study

This meta-analysis is the first to comprehensively evaluate esketamine’s perioperative effects in laparoscopic surgery patients. Previous reviews have limitations—Xie et al. (2023) assessed only postoperative pain in abdominal surgery, while Wang X. et al. (2021) and Juan et al. (2024) focused on acute and chronic pain without considering postoperative sleep. Our analysis systematically examines esketamine’s effects on perioperative heart rate, mean arterial pressure, urine output, fluid intake, blood loss, awakening time, Ramsay and SAS scores, PCIA press counts, extubation time, PACU stay, anesthesia duration, morphine consumption, hospital stay, and serum IL-6 levels. Notably, it is also the first to evaluate esketamine’s impact on postoperative fatigue in this population. By providing a more comprehensive assessment of esketamine’s perioperative safety and efficacy, this review offers a broader and more objective evaluation, making it more valuable than previous meta-analyses.

### 4.3 Limitations

Our findings suggest that esketamine does not influence perioperative morphine consumption in laparoscopic surgery patients; however, the reliability of this conclusion is uncertain due to potential biases from data extraction and human factors. Therefore, the effect of esketamine on morphine consumption remains inconclusive. The analysis of secondary outcomes is limited by the inclusion of only two to three studies, highlighting the need for more RCTs to explore these outcomes more comprehensively. Although studies such as Wen et al. (2025) have examined esketamine’s effects on perioperative depression, this review did not address this outcome. Postoperative pain was assessed using subjective scales (VAS and NRS), while sleep quality was evaluated with AIS. However, subjective pain assessments may not fully capture actual pain levels due to individual variability. PSG, the gold standard for sleep assessment, is rarely used in clinical practice due to its cost, complexity, and potential disruption to sleep (Chinoy et al., 2021; Gao et al., 2022). Although PSQI is a reliable tool, few studies have examined esketamine’s impact on postoperative sleep quality using this method. Despite these limitations, our study offers evidence supporting improvements in sleep quality from an evidence-based perspective. In this meta-analysis, substantial heterogeneity was detected between primary and secondary outcomes. Despite thorough investigation, we were unable to pinpoint the underlying sources. We postulate that this heterogeneity could be attributed to several factors: discrepancies in the definitions of various outcome metrics, the inability to conduct comprehensive subgroup analyses based on treatment administration methods or dosages, variations in the duration of outcome follow-up, and differences in sample sizes across studies. This finding further underscores the necessity of standardizing the definitions of clinically significant outcome metrics for esketamine in future research. While statistical significance is a cornerstone of research interpretation, it is crucial to recognize that it does not always equate to clinical significance. While funnel plots were provided to facilitate intuitive assessment of publication bias, quantitative tests (e.g., Egger’s regression) were not performed. This decision was based on constraints related to both the limited number of included studies and the presence of heterogeneity. Consequently, caution is warranted when interpreting potential publication bias, and the conclusions should be considered in conjunction with findings from sensitivity analyses and the overall body of evidence. This distinction underscores the importance of the minimal clinically important difference (MCID) concept—a patient-centered metric defining the smallest change in perceived benefit that outweighs associated side effects and cost burdens (Wang et al., 2023). In the context of this meta-analysis, although the VAS scores achieved statistical significance, the SMD of 0.47 falls short of the threshold typically associated with clinical relevance. Such findings highlight the need for a nuanced approach to data interpretation, emphasizing the necessity of distinguishing between statistical and clinical significance to ensure that research findings translate meaningfully into clinical practice. Lastly, a notable limitation lies in the narrow geographical scope of the included studies—specifically, only RCTs originating from China were incorporated. Such a restricted geographical focus could impose constraints on the results and undermine their ability to be generalized beyond the Chinese context. This meta-analysis focuses on esketamine’s perioperative effects from a longitudinal perspective but does not address broader clinical implications. The clinical significance of esketamine’s effects remains uncertain, and further investigation into indications and treatment optimization is needed. While meta-analyses have limited capacity to address these issues, future systematic reviews could provide more comprehensive insights.

### 4.4 Future research directions

This systematic review does not address the pharmacodynamic optimization of esketamine’s dose-response relationship in laparoscopic surgery. Future research should focus on pharmacokinetic/pharmacodynamic (PK/PD) modeling to identify interventional thresholds, particularly in dose-dependent modulation of hyperalgesia during minimally invasive procedures. Subsequent trials should establish comprehensive therapeutic indices that integrate neurocognitive recovery, long-term pain management, and pharmacoeconomic assessments of esketamine’s risk-benefit profile. Practice-changing clinical trials with adaptive Bayesian designs across tertiary referral centers could resolve the current uncertainty regarding esketamine’s role in enhanced recovery protocols. These trials should adhere to SPIRIT-COSMIN guidelines for evaluating complex interventions.

## 5 Conclusion

Esketamine demonstrates multimodal therapeutic benefits in laparoscopic surgery patients, including enhanced postoperative analgesia, reduced intraoperative opioid requirements, improved sleep architecture, mitigated postoperative fatigue, accelerated functional recovery, and elevated plasma BDNF concentrations. To further validate these conclusions, large-scale international multicenter randomized controlled trials (RCTs) should adopt standardized dosing regimens and unified outcome measures in the future.

## Data Availability

The original contributions presented in the study are included in the article/Supplementary Material, further inquiries can be directed to the corresponding author.
