# Supplementary material for: Effectiveness and safety of esketamine in laparoscopic surgery patients: a systematic review and meta-analysis of randomized controlled trials
Source: Front Pharmacol. 2025 Oct 21;16:1663348. doi: 10.3389/fphar.2025.1663348 (PMC12583057; doi:10.3389/fphar.2025.1663348)
Supplement: Supplementary file 1 [file Supplementaryfile1.docx]

# Table S1 Literature search strategy

**1.Pubmed**

| Search number | Query |
| --- | --- |
| #1 | "Esketamine" [Supplementary Concept] 659 |
| #2 | (((((((((((((((((((((L-Ketamine[Title/Abstract]) OR (S-Ketamine[Title/Abstract])) OR (Kataved[Title/Abstract])) OR (Spravato[Title/Abstract])) OR (am 101[Title/Abstract])) OR (cle 100[Title/Abstract])) OR (cz 06[Title/Abstract])) OR (esgamda[Title/Abstract])) OR (eskelan[Title/Abstract])) OR (esketamin[Title/Abstract])) OR (esketamine hydrochloride[Title/Abstract])) OR (esketiv[Title/Abstract])) OR (falkieri[Title/Abstract])) OR (jnj 54135419[Title/Abstract])) OR (jnj 5419[Title/Abstract])) OR (ketanest s[Title/Abstract])) OR (keyzilen[Title/Abstract])) OR (pg 061[Title/Abstract])) OR (s-ketamin[Title/Abstract])) OR (sinmelan[Title/Abstract])) OR (vesierra[Title/Abstract])) OR (esketamine[Title/Abstract]) 6723 |
| #3 | "Laparoscopy"[Mesh] 124082 |
| #4 | (((((Laparoscopy*[Title/Abstract]) OR (Peritoneoscopy*[Title/Abstract])) OR (Celioscopy*[Title/Abstract])) OR (Laparoscopic Assisted Surgery*[Title/Abstract])) OR (Laparoscopic Surgical Procedure*[Title/Abstract])) OR (Laparoscopic Surgery*[Title/Abstract]) 68722 |
| #5 | ("Laparoscopy"[Mesh]) OR ((((((Laparoscopy*[Title/Abstract]) OR (Peritoneoscopy*[Title/Abstract])) OR (Celioscopy*[Title/Abstract])) OR (Laparoscopic Assisted Surgery*[Title/Abstract])) OR (Laparoscopic Surgical Procedure*[Title/Abstract])) OR (Laparoscopic Surgery*[Title/Abstract])) 149348 |
| #6 | ("Esketamine" [Supplementary Concept]) OR ((((((((((((((((((((((L-Ketamine[Title/Abstract]) OR (S-Ketamine[Title/Abstract])) OR (Kataved[Title/Abstract])) OR (Spravato[Title/Abstract])) OR (am 101[Title/Abstract])) OR (cle 100[Title/Abstract])) OR (cz 06[Title/Abstract])) OR (esgamda[Title/Abstract])) OR (eskelan[Title/Abstract])) OR (esketamin[Title/Abstract])) OR (esketamine hydrochloride[Title/Abstract])) OR (esketiv[Title/Abstract])) OR (falkieri[Title/Abstract])) OR (jnj 54135419[Title/Abstract])) OR (jnj 5419[Title/Abstract])) OR (ketanest s[Title/Abstract])) OR (keyzilen[Title/Abstract])) OR (pg 061[Title/Abstract])) OR (s-ketamin[Title/Abstract])) OR (sinmelan[Title/Abstract])) OR (vesierra[Title/Abstract])) OR (esketamine[Title/Abstract])) 6738 |
| #7 | (("Esketamine" [Supplementary Concept]) OR ((((((((((((((((((((((L-Ketamine[Title/Abstract]) OR (S-Ketamine[Title/Abstract])) OR (Kataved[Title/Abstract])) OR (Spravato[Title/Abstract])) OR (am 101[Title/Abstract])) OR (cle 100[Title/Abstract])) OR (cz 06[Title/Abstract])) OR (esgamda[Title/Abstract])) OR (eskelan[Title/Abstract])) OR (esketamin[Title/Abstract])) OR (esketamine hydrochloride[Title/Abstract])) OR (esketiv[Title/Abstract])) OR (falkieri[Title/Abstract])) OR (jnj 54135419[Title/Abstract])) OR (jnj 5419[Title/Abstract])) OR (ketanest s[Title/Abstract])) OR (keyzilen[Title/Abstract])) OR (pg 061[Title/Abstract])) OR (s-ketamin[Title/Abstract])) OR (sinmelan[Title/Abstract])) OR (vesierra[Title/Abstract])) OR (esketamine[Title/Abstract]))) AND (("Laparoscopy"[Mesh]) OR ((((((Laparoscopy*[Title/Abstract]) OR (Peritoneoscopy*[Title/Abstract])) OR (Celioscopy*[Title/Abstract])) OR (Laparoscopic Assisted Surgery*[Title/Abstract])) OR (Laparoscopic Surgical Procedure*[Title/Abstract])) OR (Laparoscopic Surgery*[Title/Abstract]))) 42 |

**2.Cochrane**

| Search number | Query |
| --- | --- |
| #1 | MeSH descriptor: [Laparoscopy] explode all trees 9445 |
| #2 | (L-Ketamine OR S-Ketamine OR Kataved OR Spravato OR am 101 OR cle 100 OR cz 06 OR esgamda OR eskelan OR esketamin OR esketamine hydrochloride OR esketiv OR falkieri OR jnj 54135419 OR jnj 5419 OR ketanest s OR keyzilen OR pg 061 OR s-ketamin OR sinmelan OR vesierra OR esketamine):ti,ab,kw (Word variations have been searched) 2372 |
| #3 | (Laparoscopy* OR Peritoneoscopy* OR Celioscopy* OR Laparoscopic Assisted Surgery* OR Laparoscopic Surgical Procedure* OR Laparoscopic Surgery* OR pelvic endoscopy):ti,ab,kw (Word variations have been searched) 25282 |
| #4 | #1 OR #3 25861 |
| #5 | #2 AND #4 156 |

**3.Embase**

| Search number | Query |
| --- | --- |
| #1 | 'esketamine'/exp OR 'esketamine' 2512 |
| #2 | 'laparoscopy'/exp OR 'laparoscopy' 237558 |
| #3 | laparoscopy*:ab,ti OR peritoneoscopy*:ab,ti OR celioscopy*:ab,ti OR 'laparoscopic assisted surgery*':ab,ti OR 'laparoscopic surgical procedure*':ab,ti OR 'laparoscopic surgery*':ab,ti OR 'pelvic endoscopy':ab,ti 93449 |
| #4 | 'l ketamine':ab,ti OR 's ketamine':ab,ti OR kataved:ab,ti OR pravato:ab,ti OR 'am 101':ab,ti OR 'cle 100':ab,ti OR 'cz 06':ab,ti OR esgamda:ab,ti OR eskelan:ab,ti OR esketamin:ab,ti OR 'esketamine hydrochloride':ab,ti OR esketiv:ab,ti OR falkieri:ab,ti OR 'jnj 54135419':ab,ti OR 'jnj 5419':ab,ti OR 'ketanest s':ab,ti OR keyzilen:ab,ti OR 'pg 061':ab,ti OR 's ketamin':ab,ti OR sinmelan:ab,ti OR vesierra:ab,ti OR esketamine:ab,ti 2404 |
| #5 | #1 OR #4 3204 |
| #6 | #2 OR #3 240846 |
| #7 | #5 AND #6 66 |

**4.Web of science**

| Search number | Query |
| --- | --- |
| #1 | TS=(L-Ketamine OR S-Ketamine OR Kataved OR Spravato OR am 101 OR cle 100 OR cz 06 OR esgamda OR eskelan OR esketamin OR esketamine hydrochloride OR esketiv OR falkieri OR jnj 54135419 OR jnj 5419 OR ketanest s OR keyzilen OR pg 061 OR s-ketamin OR sinmelan OR vesierra OR esketamine) 1019 |
| #2 | TS=(Laparoscopy* OR Peritoneoscopy* OR Celioscopy* OR Laparoscopic Assisted Surgery* OR Laparoscopic Surgical Procedure* OR Laparoscopic Surgery* OR pelvic endoscopy) 2126 |
| #3 | #2 AND #1 0 |

**5.** **China Biology Medicine Database**

| Search number | Query |
| --- | --- |
| #1 | "艾司氯胺酮"[常用字段:智能] 1094 |
| #2 | "腹腔镜手术"[常用字段:智能] OR "腹腔镜外科"[常用字段:智能] 255236 |
| #3 | ("腹腔镜手术"[常用字段:智能] OR "腹腔镜外科"[常用字段:智能]) AND ("艾司氯胺酮"[常用字段:智能]) 84 |

**6.VIP**

(摘要=艾司氯胺酮 AND ((((摘要=腹腔镜手术 OR 摘要=laparoscopic operation) OR 摘要=laparoscopic surgery) OR 摘要=laparoscopic surgical procedures) OR 摘要=腹腔镜外科)) 54

**7.CNKI**

（主题：艾司氯胺酮） AND （主题：腹腔镜手术+laparoscopic operation + laparoscopic surgery + laparoscopic surgical procedures + 腹腔镜外科）235

**8.Wanfang**

主题:(腹腔镜手术 OR laparoscopic operation OR laparoscopic surgery OR laparoscopic surgical procedures OR 腹腔镜外科) and 主题:(艾司氯胺酮) 157

**Supplementary Figures**


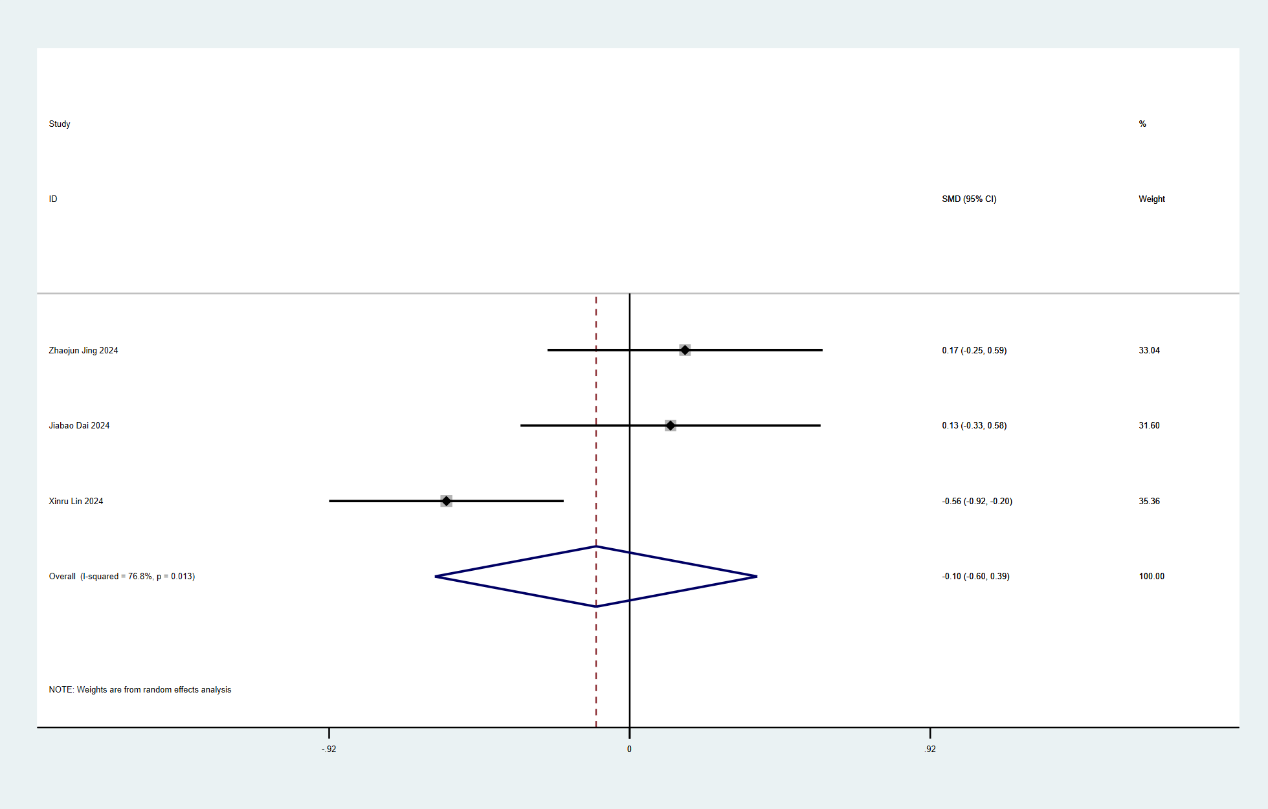


Figure S1:Forest plot of VAS scores at 2-3 days postoperatively


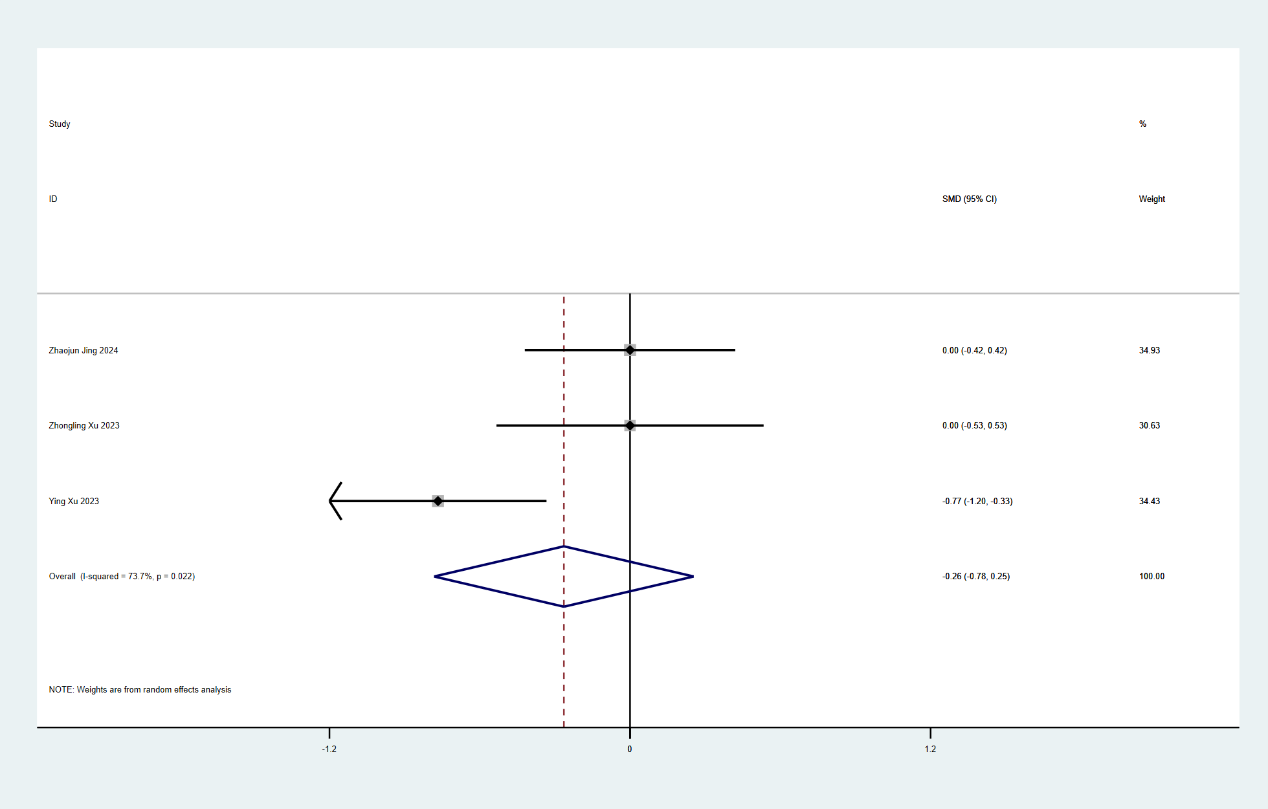


Figure S2:Forest plot of VAS scores during postoperative coughing


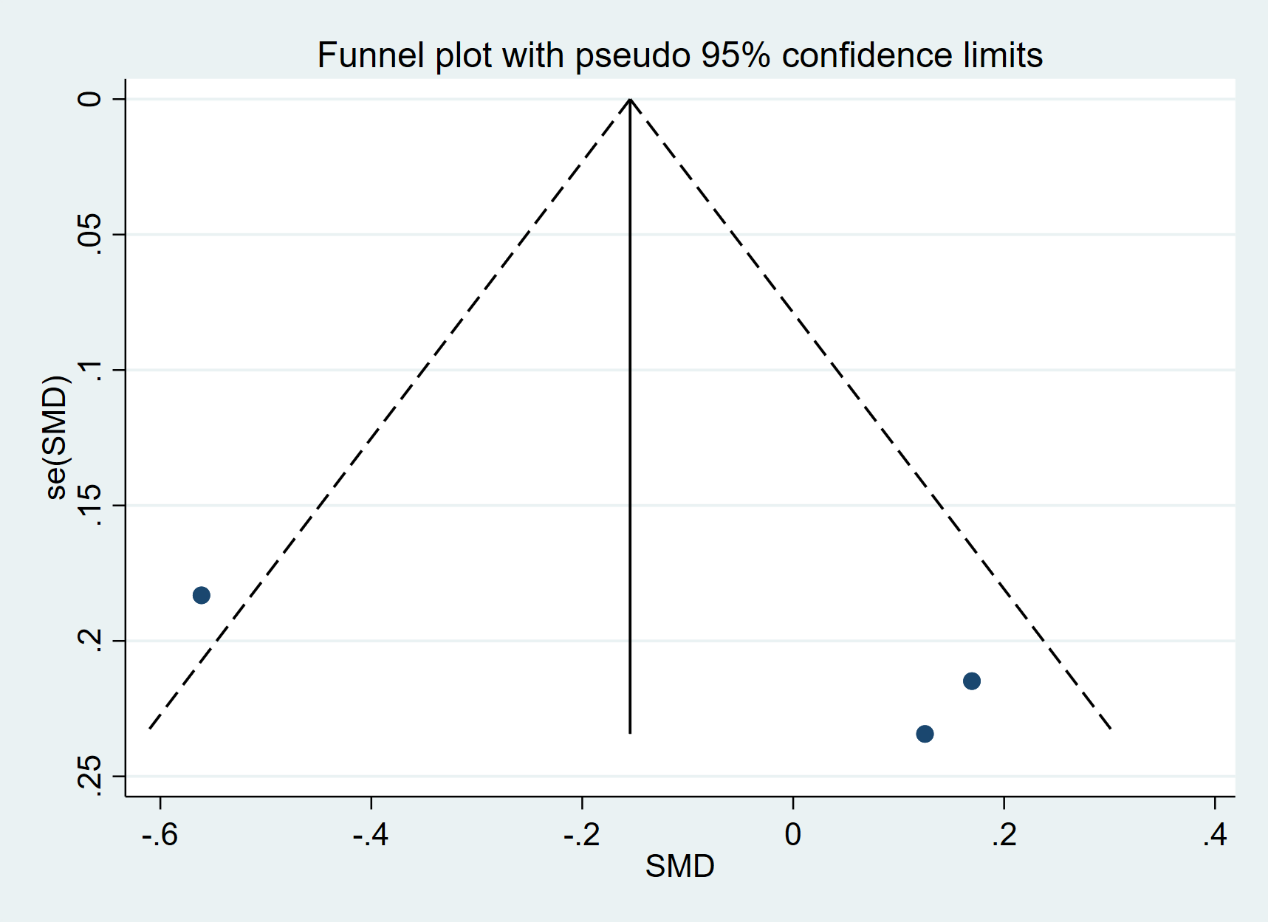


Figure S3:Funnel plot of VAS scores at rest 2-3 days after surgery


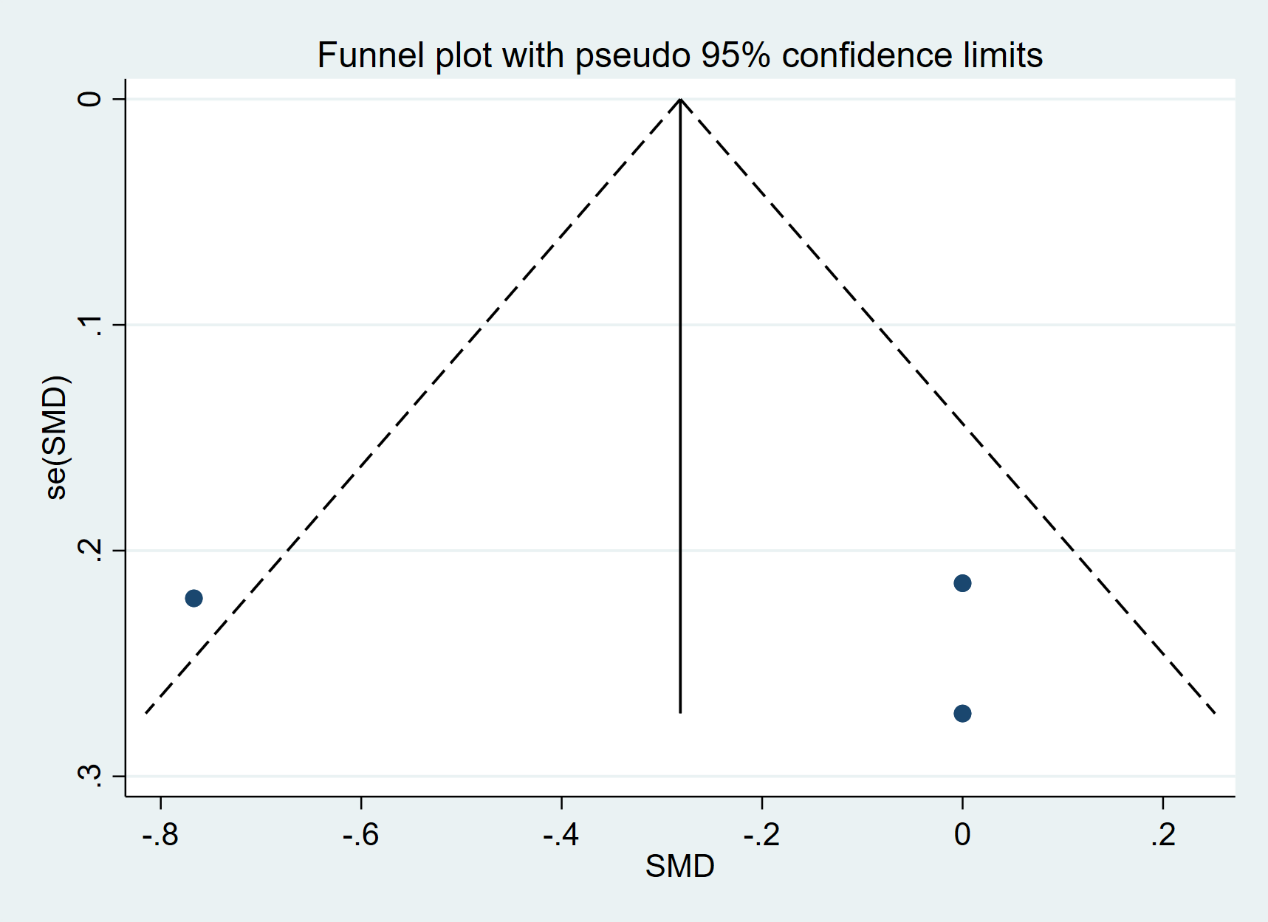


Figure S4:Funnel plot of VAS score during postoperative coughing


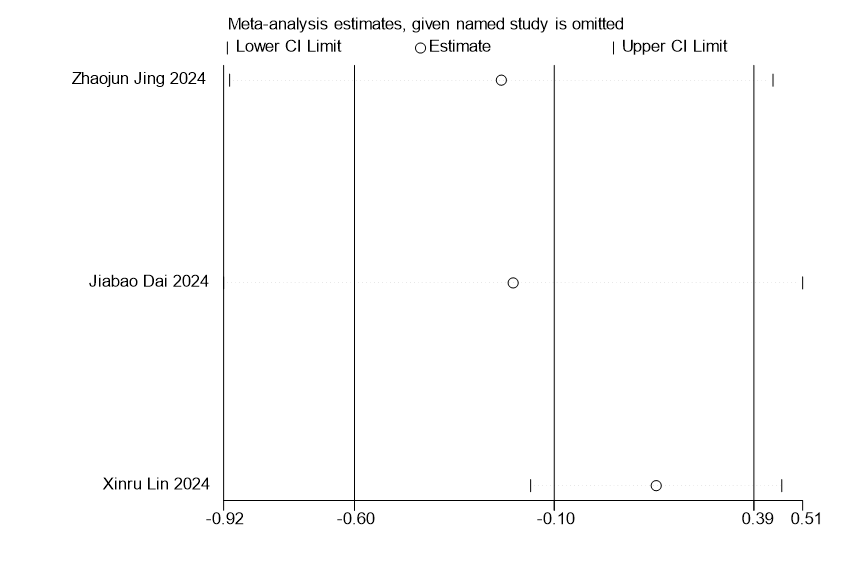


Figure S5:Sensitivity analysis of VAS scores at rest 2-3 days after surgery


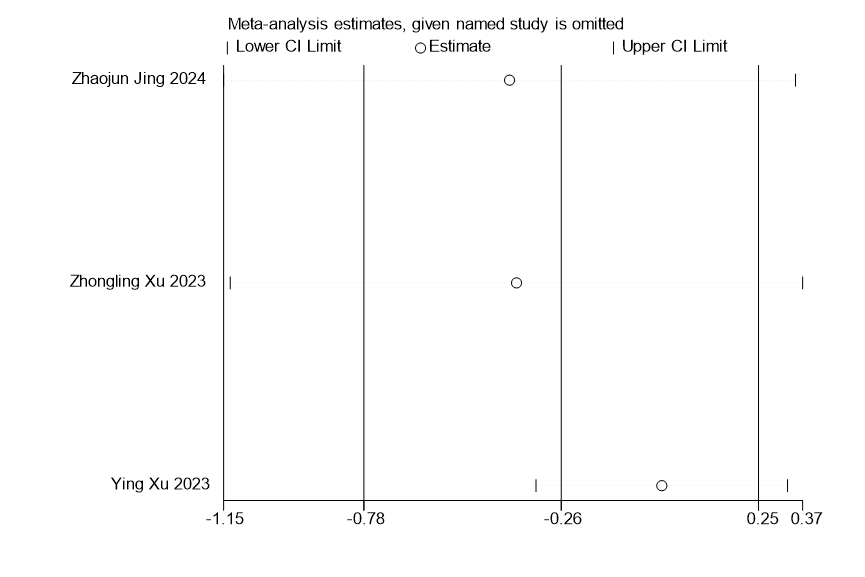


Figure S6:Sensitivity analysis of VAS scores during postoperative coughing


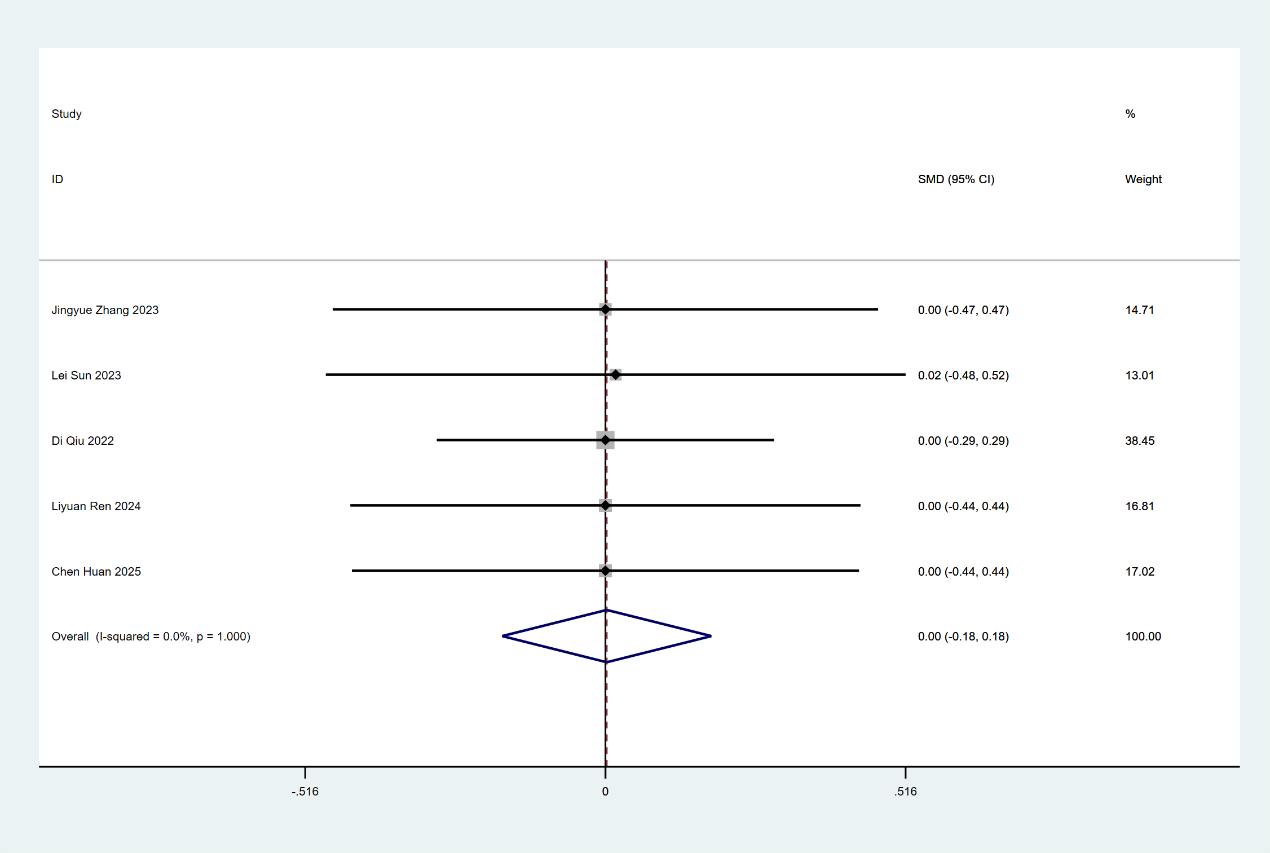


Figure S7: Forest plot of NRS scores at 1 day postoperatively


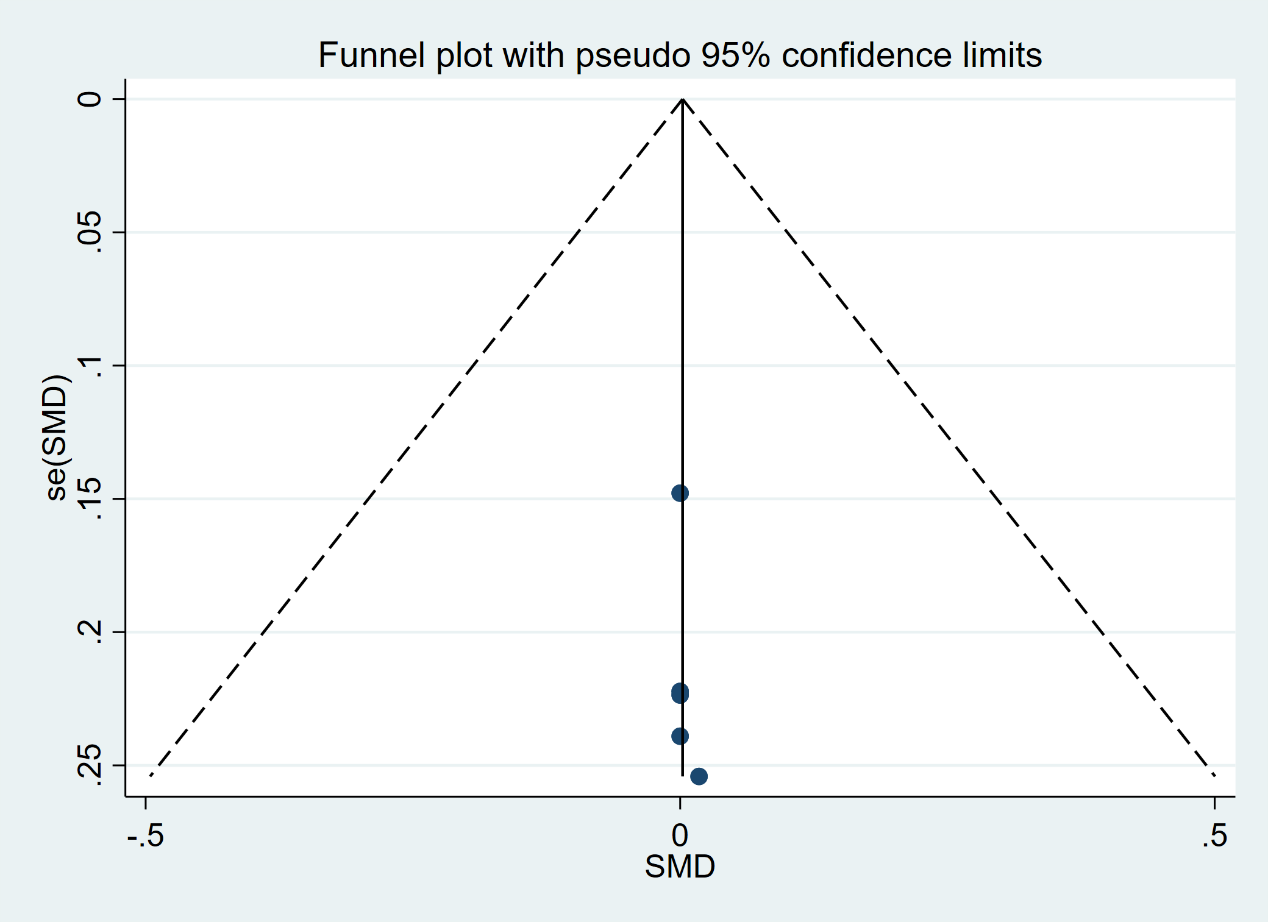


Figure S8: Funnel plot of NRS scores at 1 day postoperatively


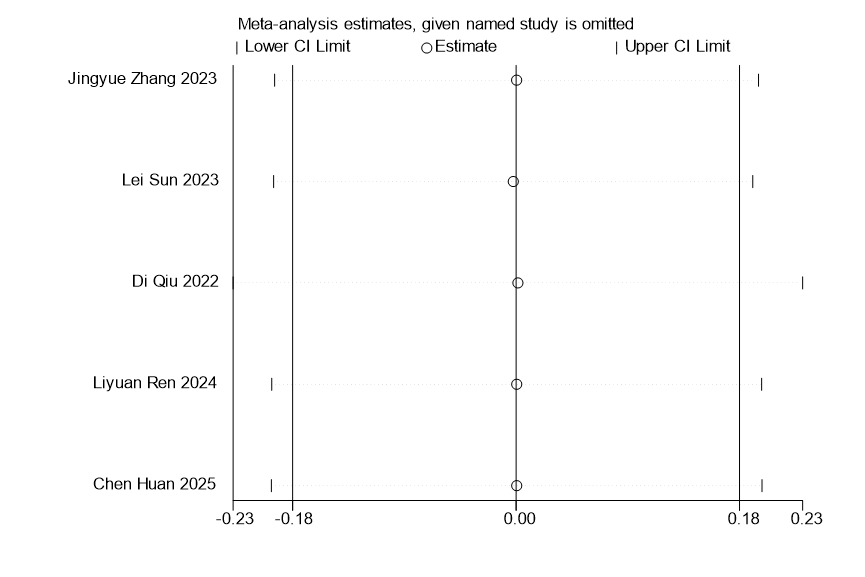


Figure S9: Sensitivity analysis of NRS scores at 1 day postoperatively


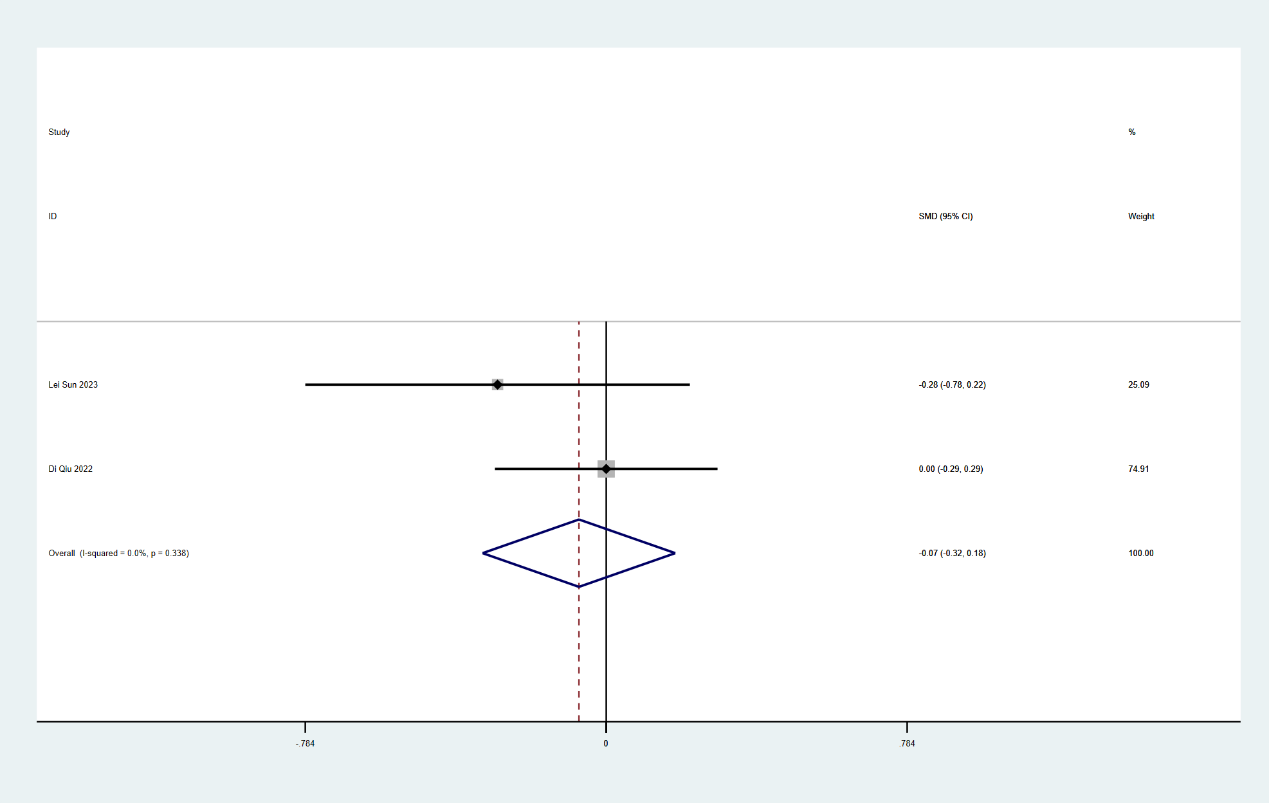


Figure S10: Forest plot of NRS scores at 3 days postoperatively


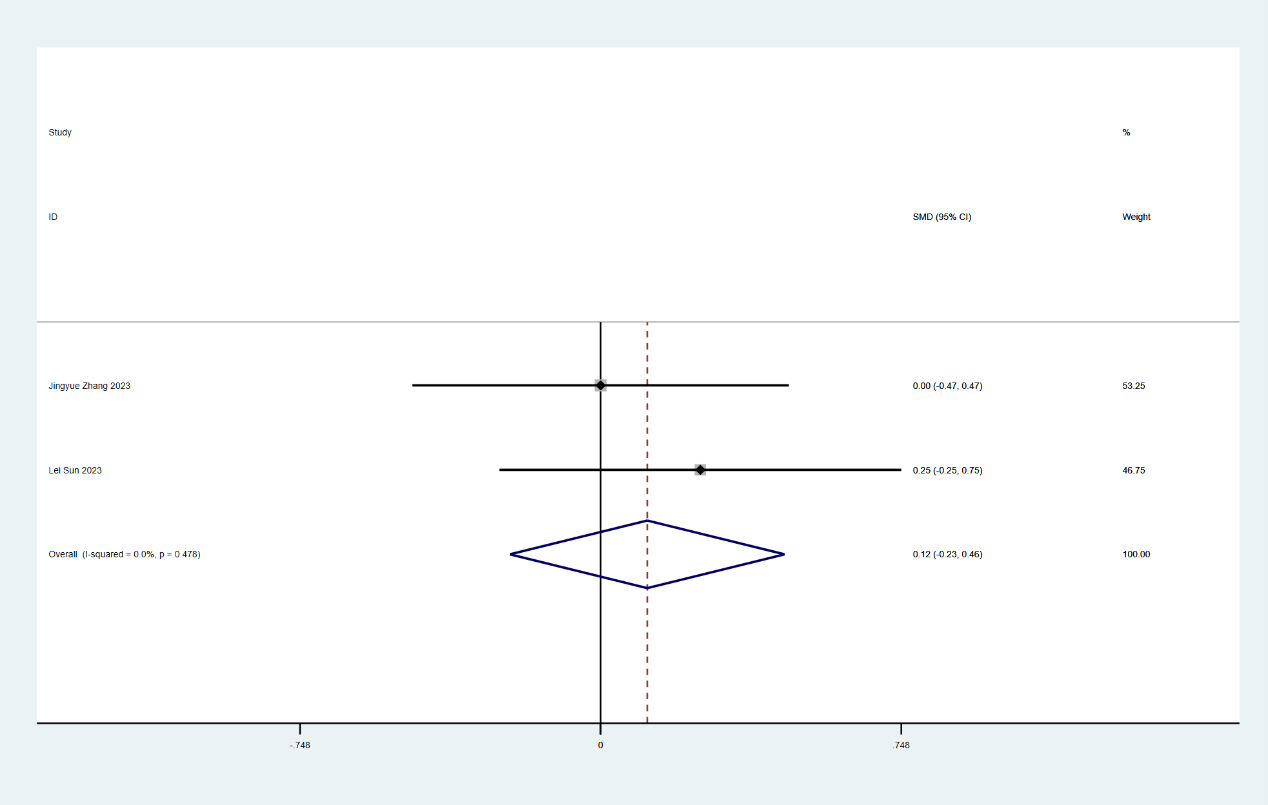


Figure S11: Forest plot of NRS scores at 7 days postoperatively


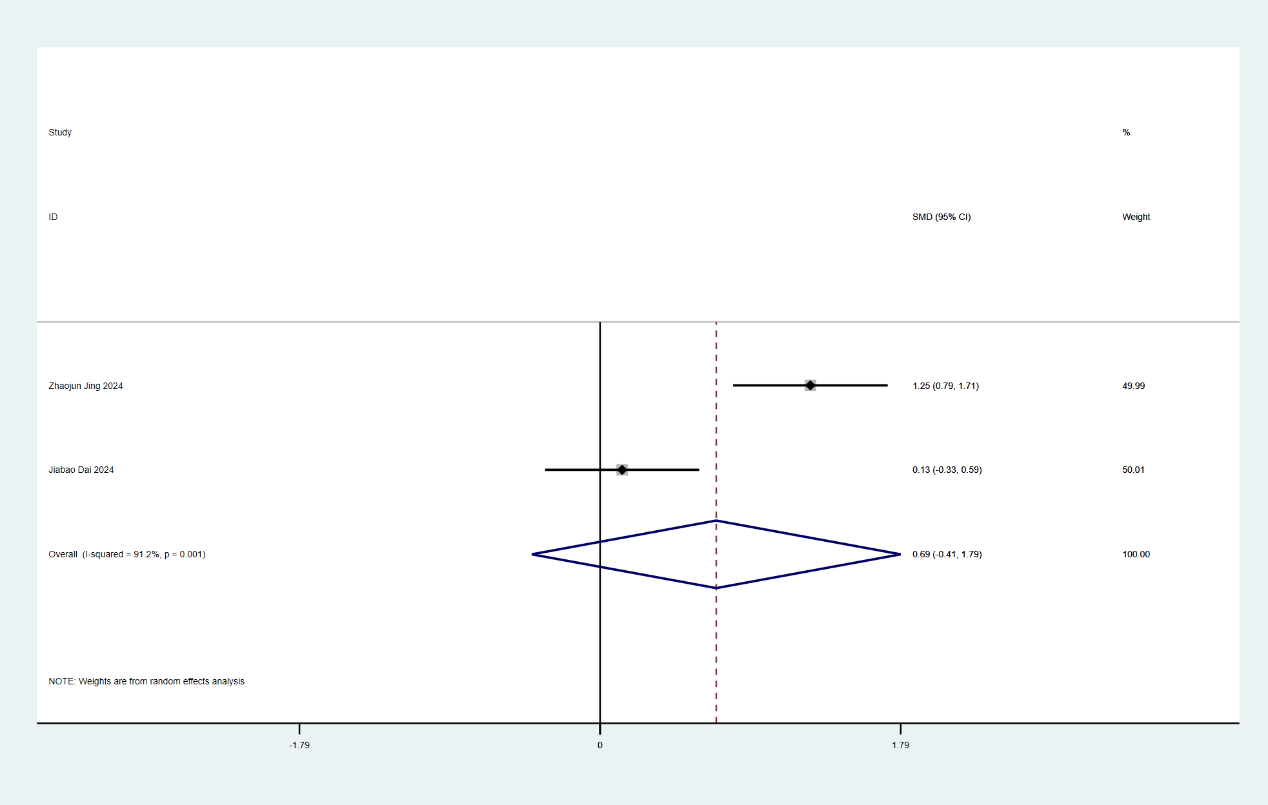


Figure S12: Forest plot of QoR-15 score at 2 days postoperatively


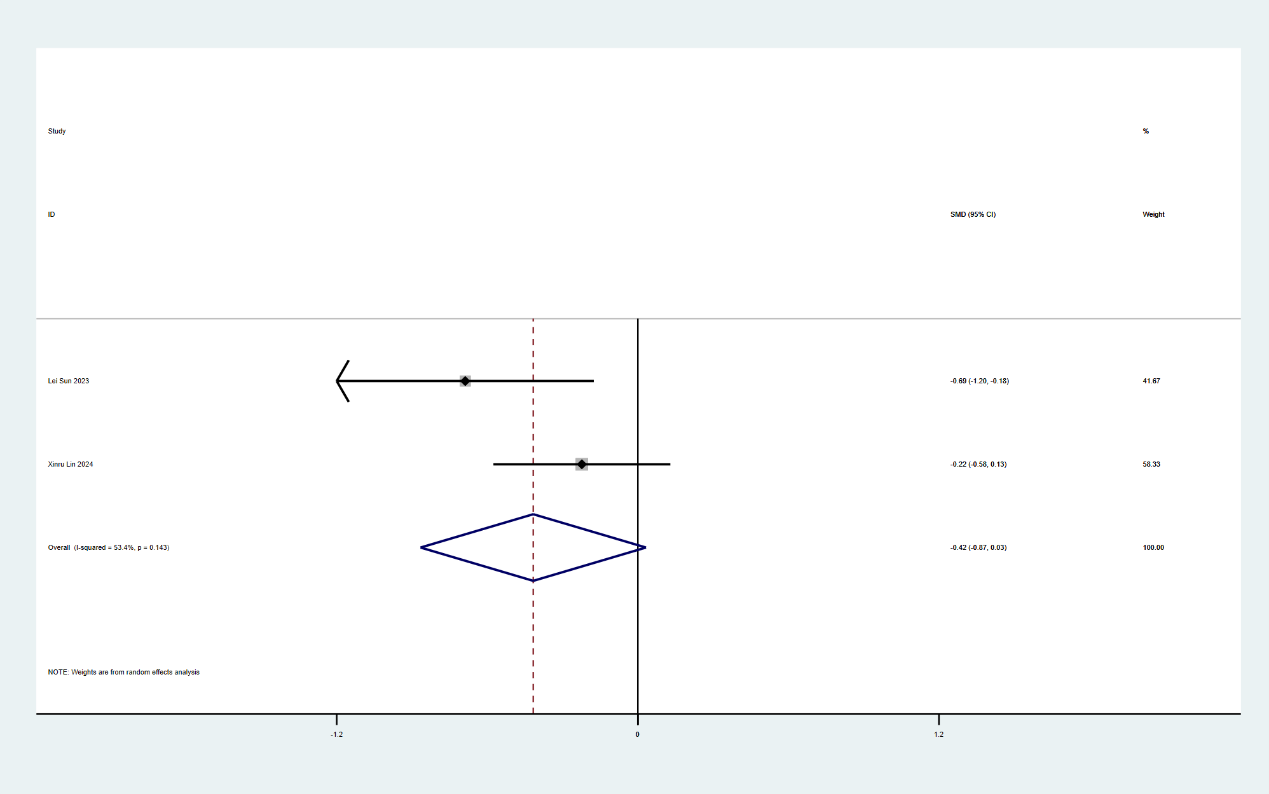


Figure S13: Forest plot of ICFS-10 score at 7 days postoperatively


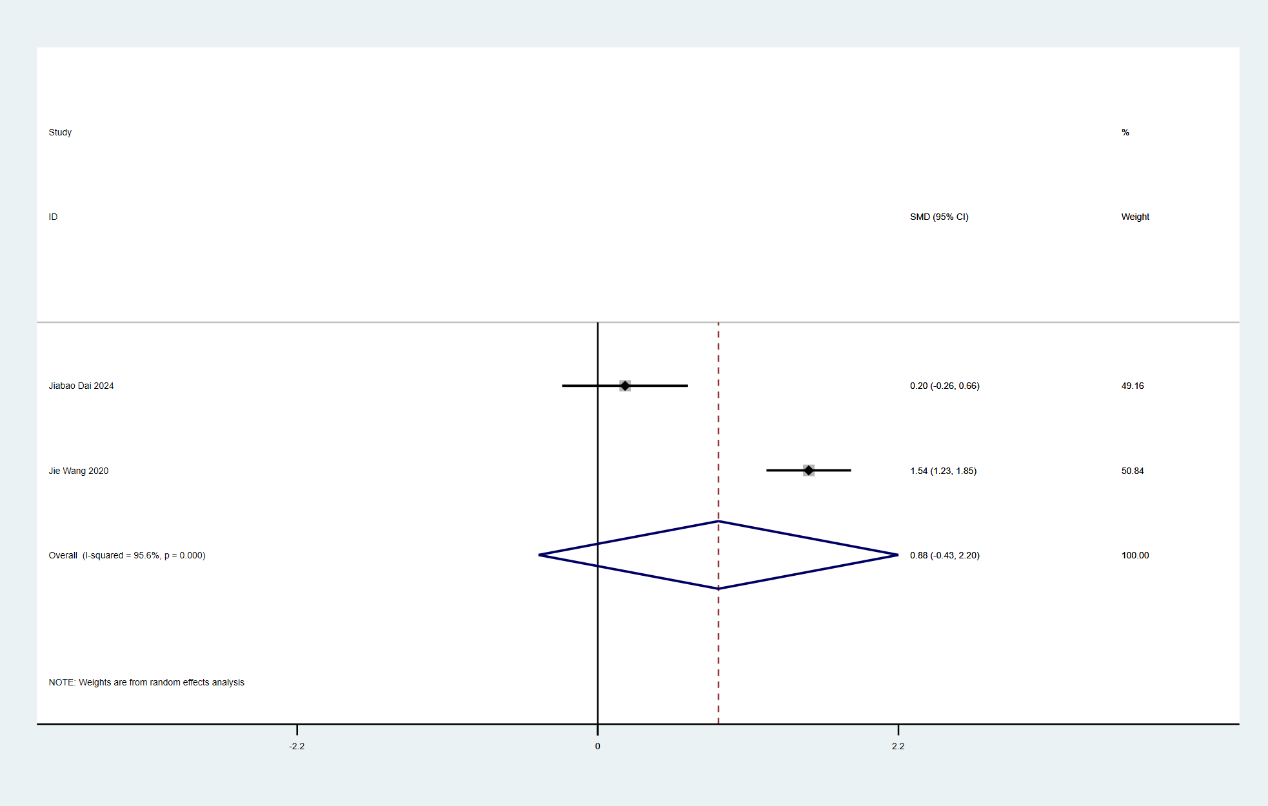


Figure S14: Forest plot of BDNF factor concentrations at 1 day postoperatively


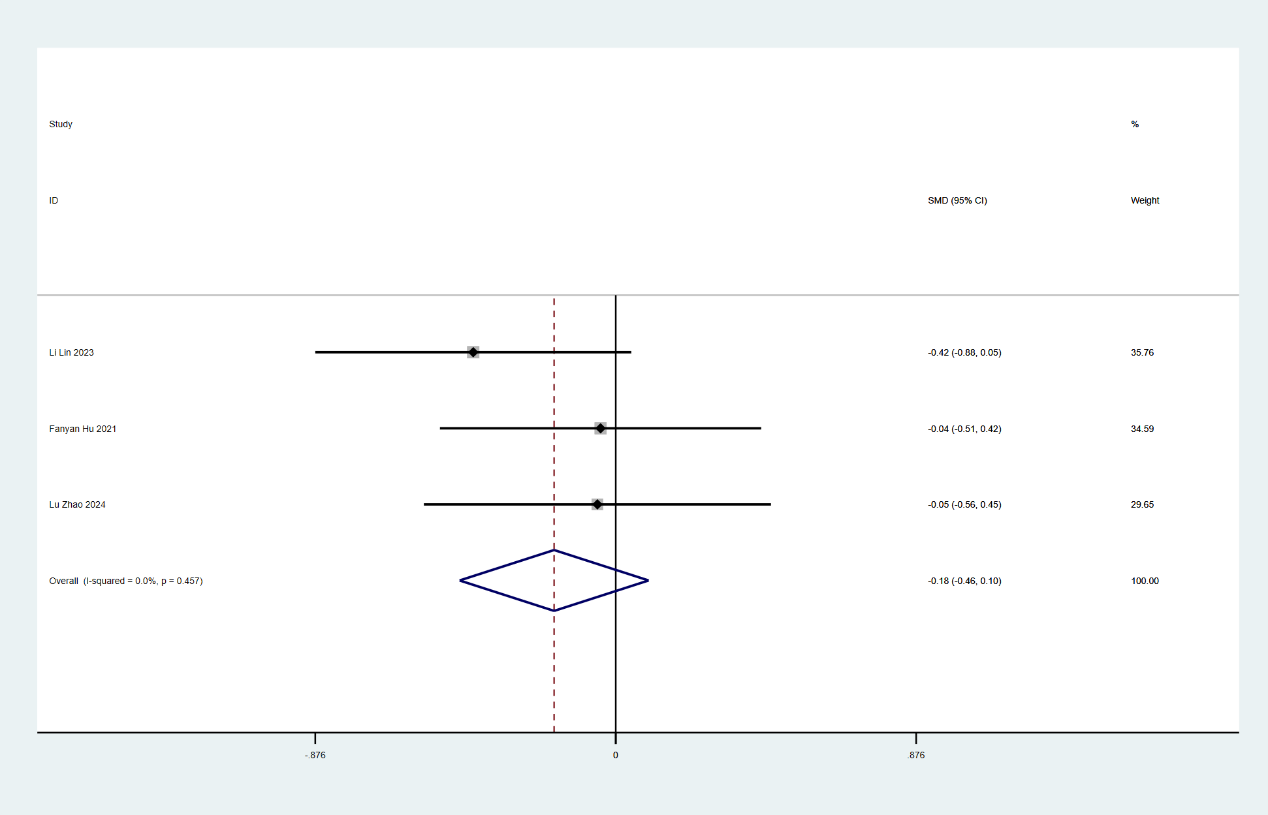


Figure S15: Forest plot of the effect of esketamine on postoperative heart rate


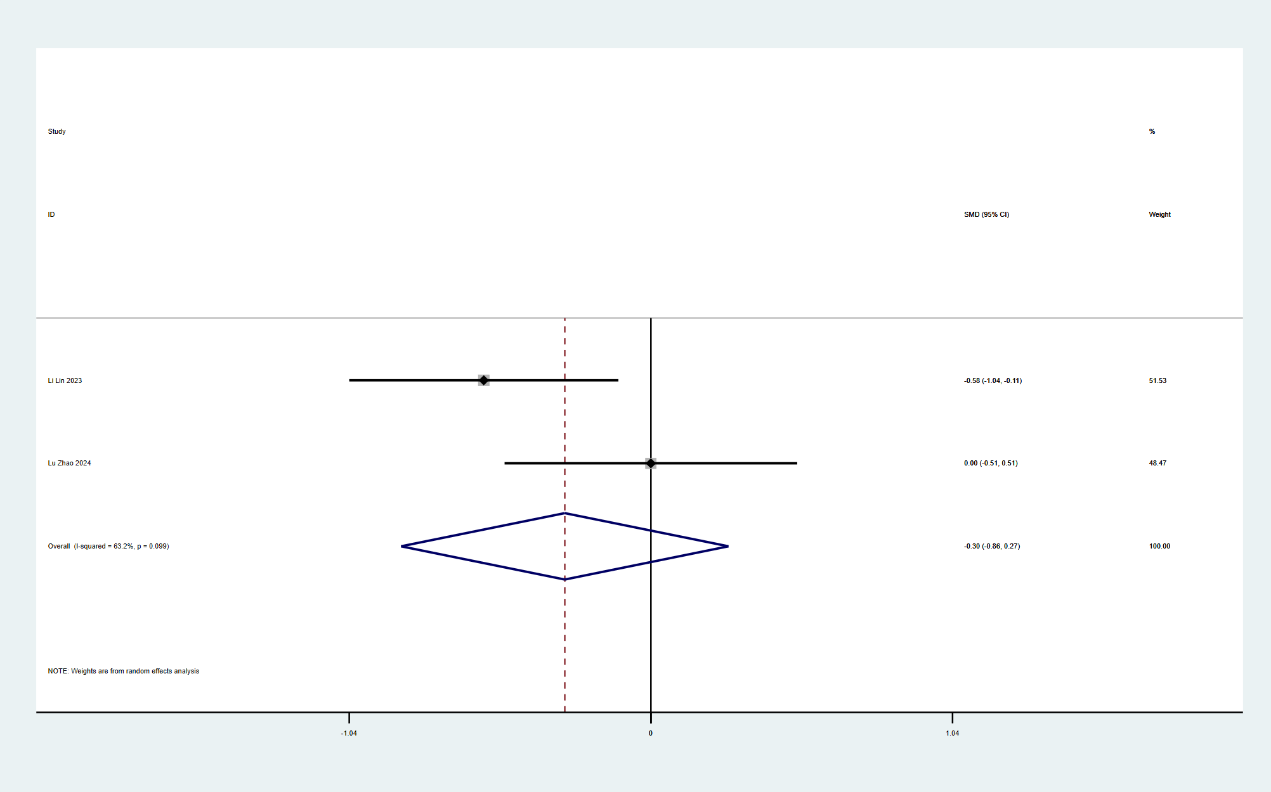


Figure S16: Forest plot of the effect of esketamine on postoperative mean arterial pressure


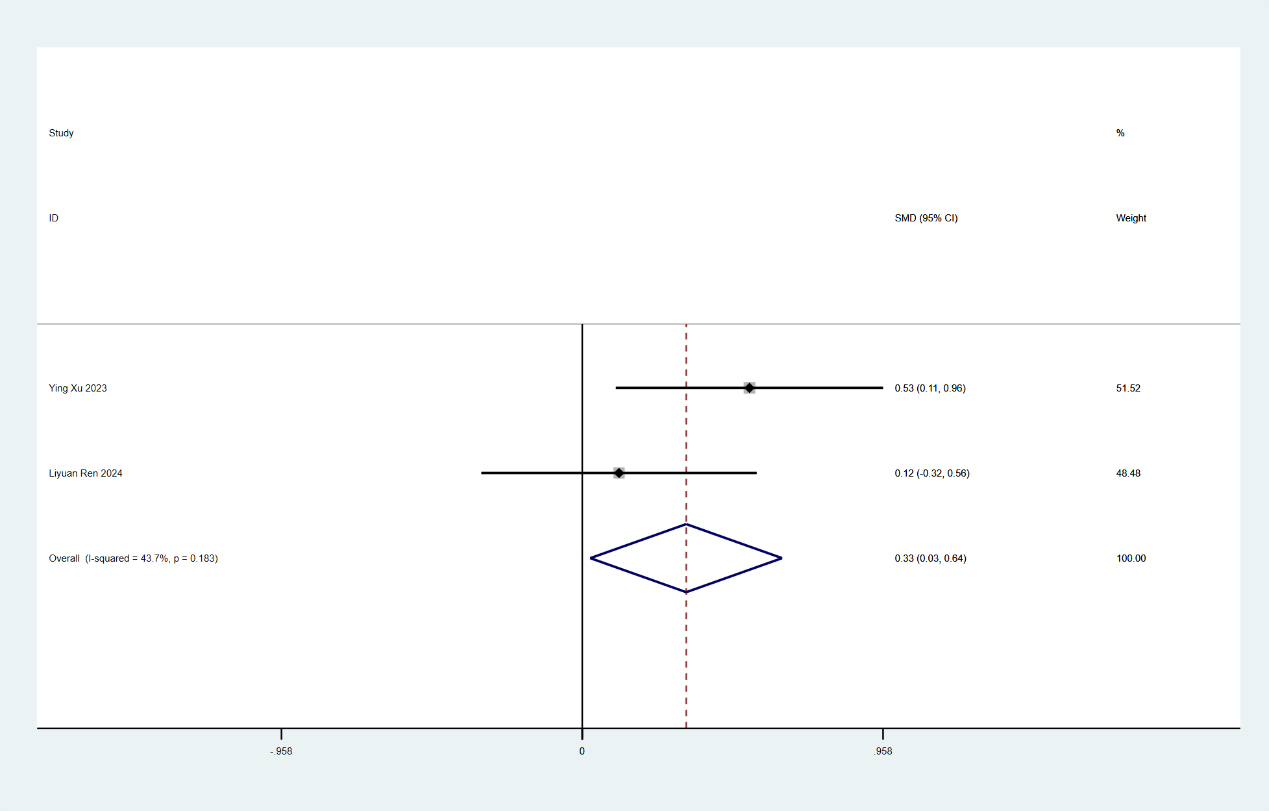


Figure S17: Forest plot of the effect of esketamine on intraoperative urine output


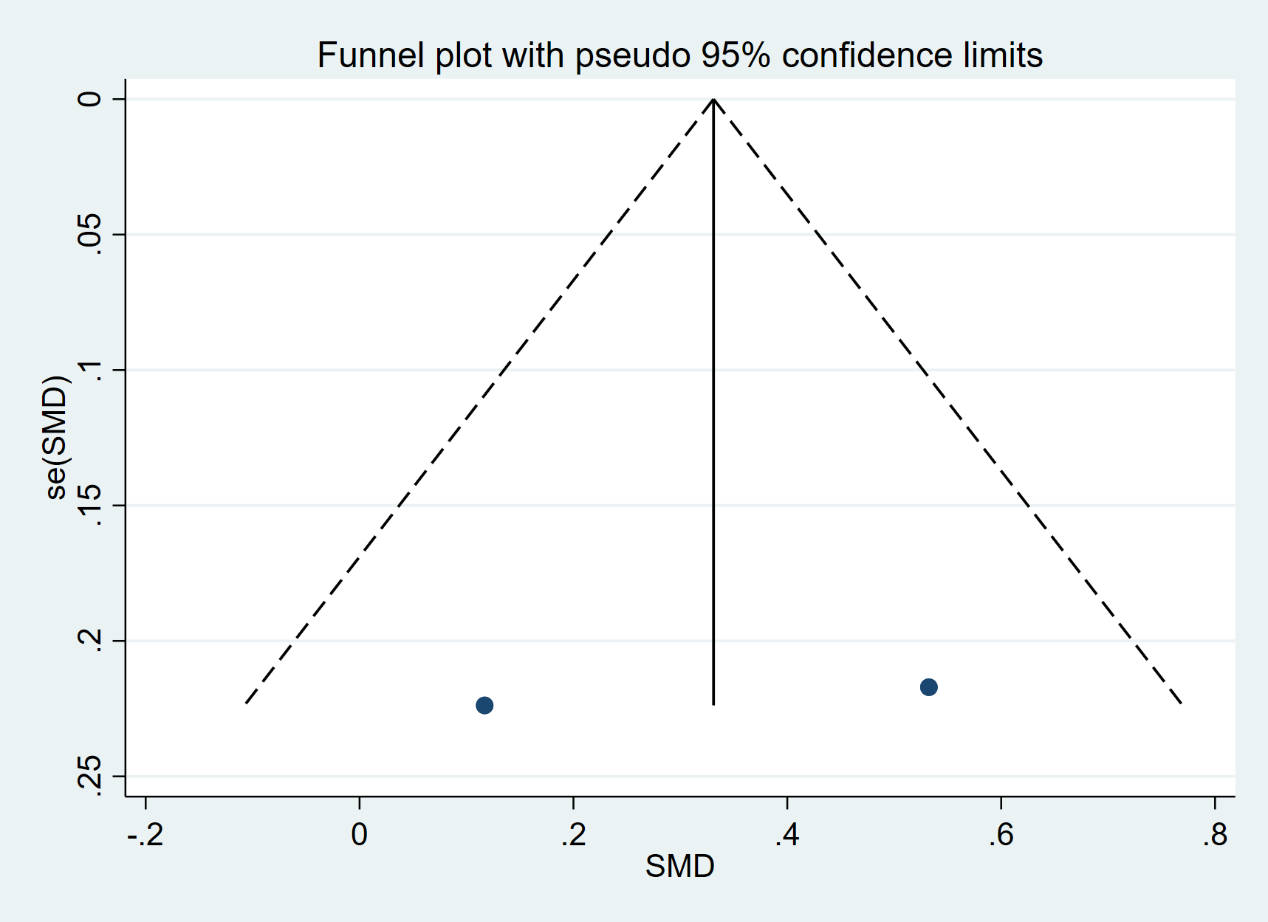


Figure S18: Funnel plot of the effect of esketamine on intraoperative urine output


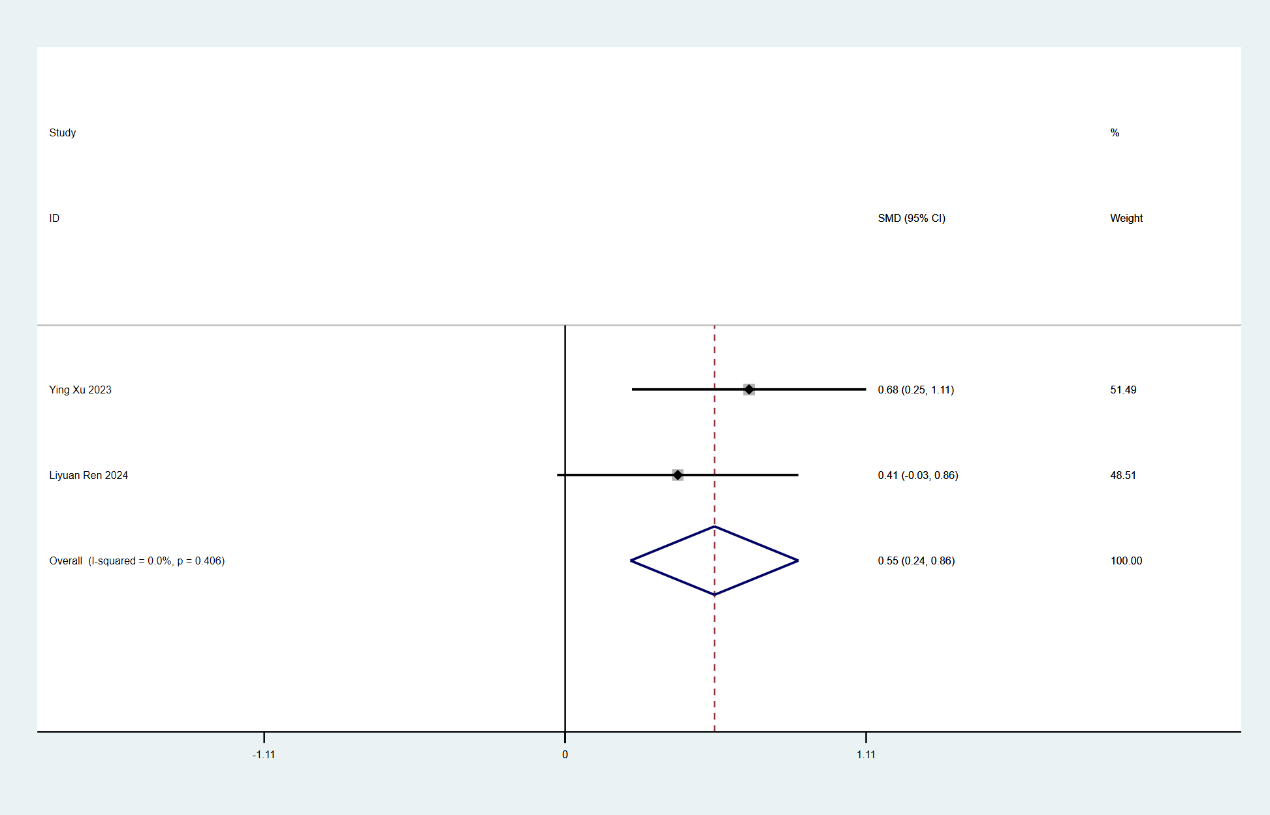


Figure S19: Forest plot of the effect of esketamine on intraoperative infusion volume


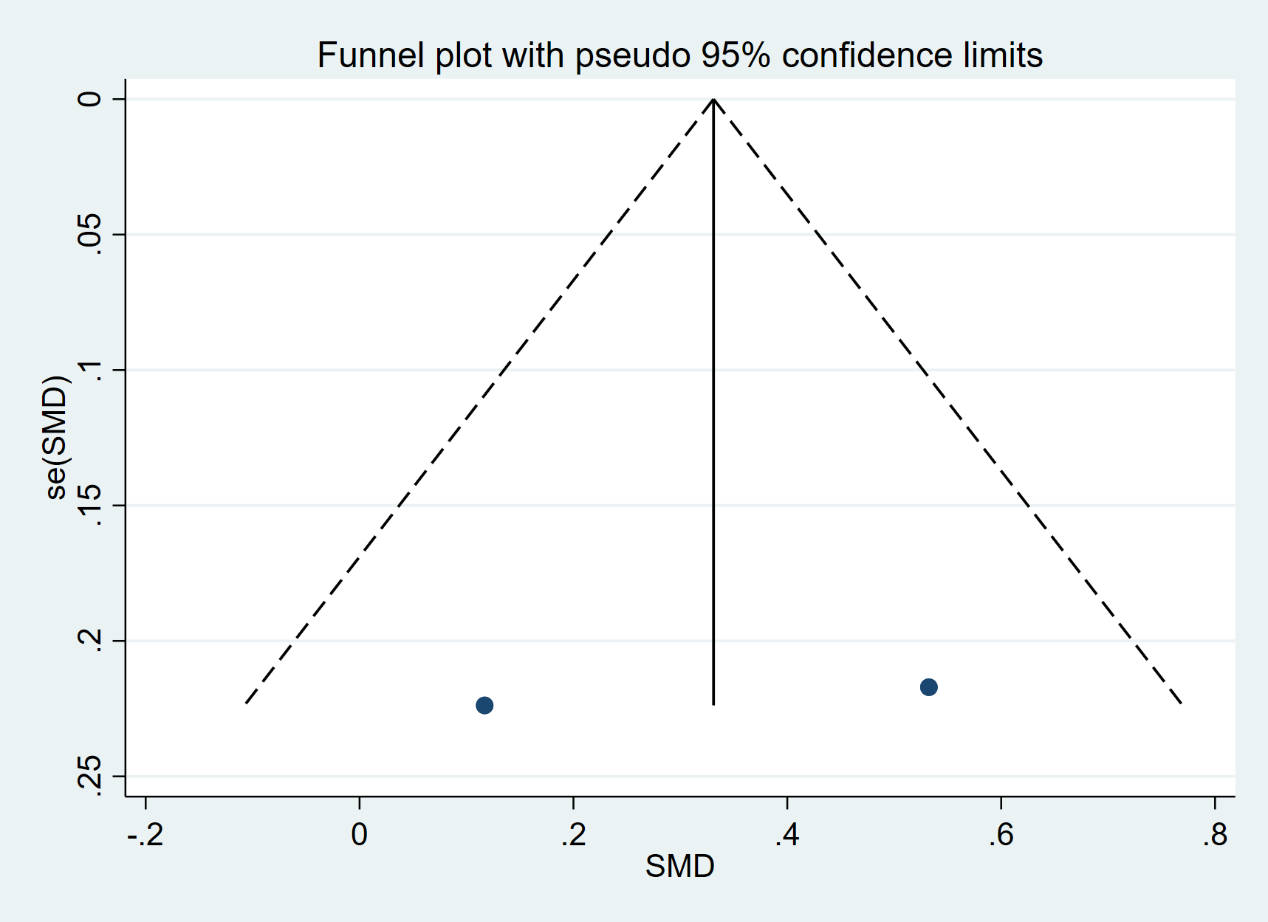


Figure S20: Funnel plot of the effect of esketamine on intraoperative infusion volume


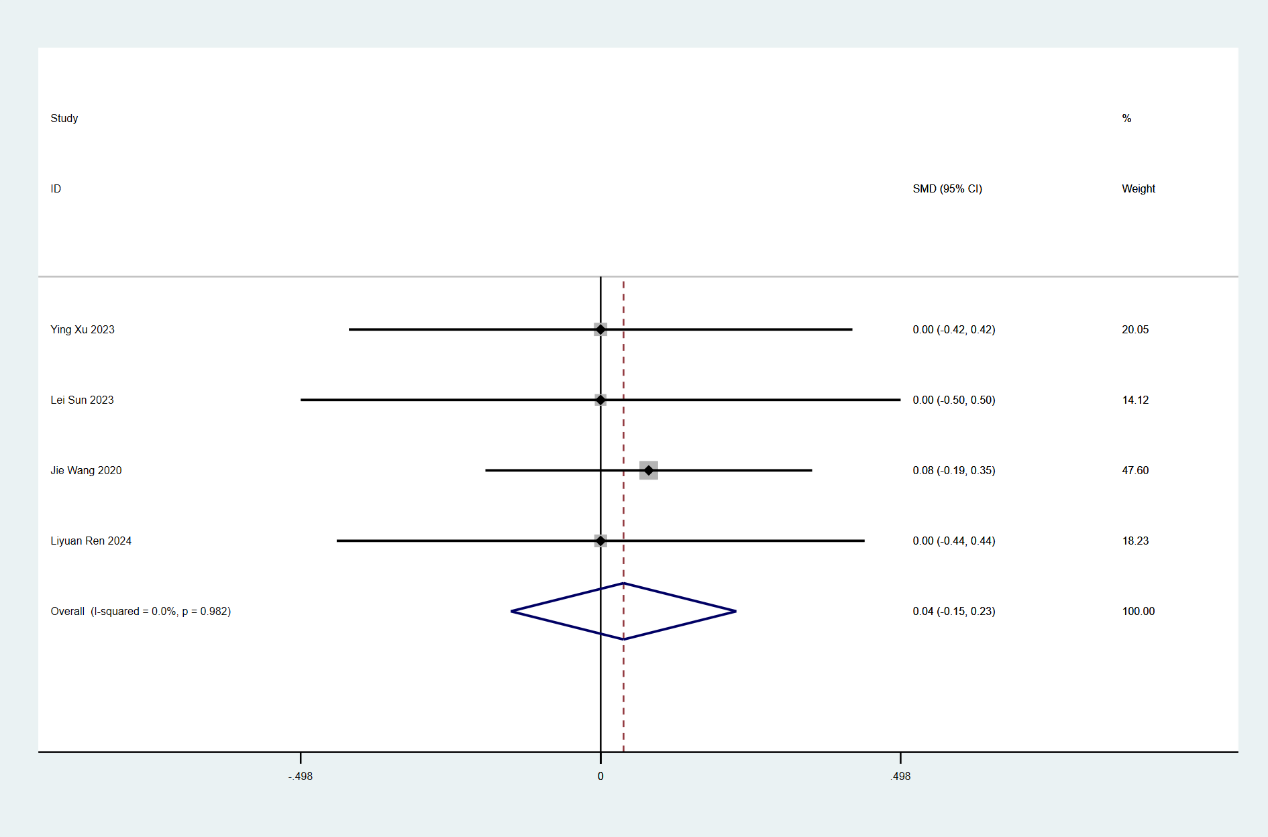


Figure S21: Forest plot of the effect of esketamine on intraoperative blood loss


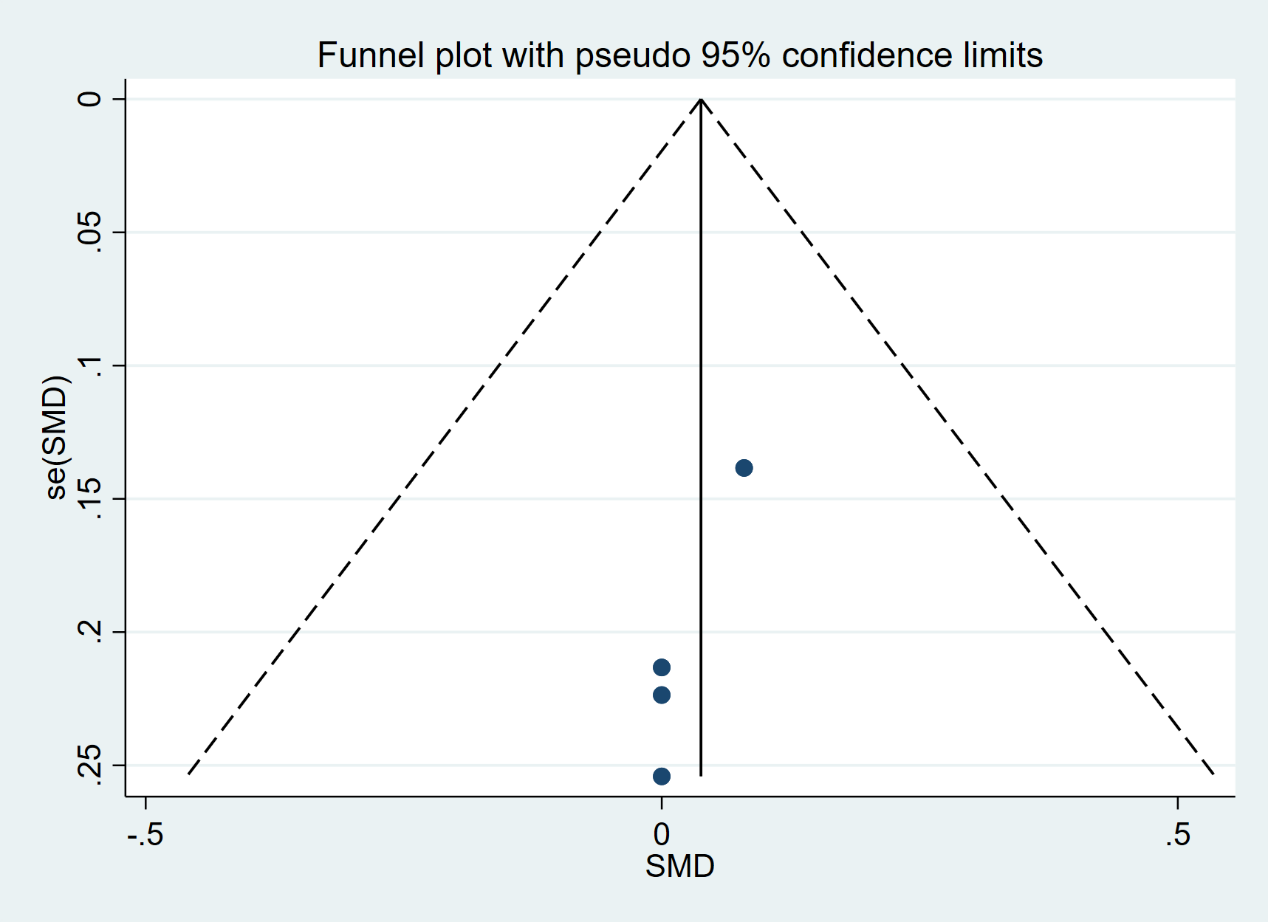


Figure S22: Funnel plot of the effect of esketamine on intraoperative blood loss


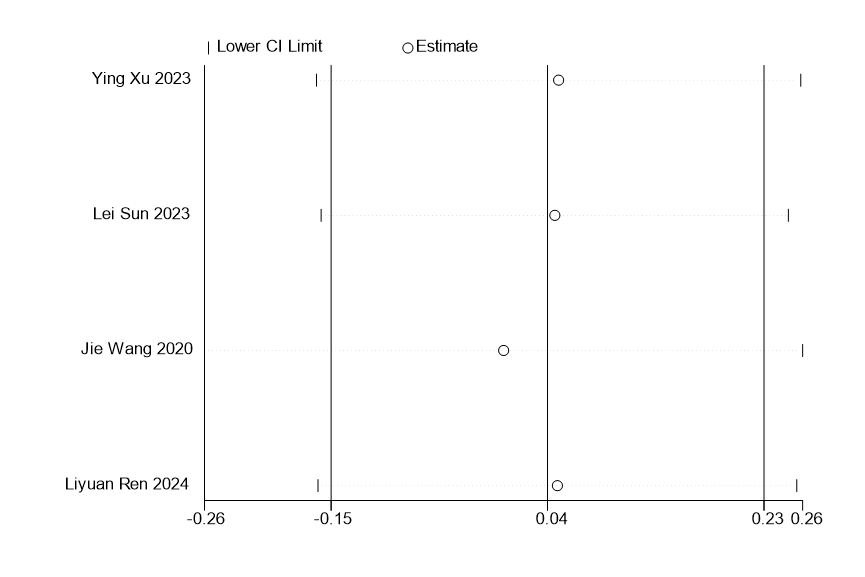


Figure S23: Sensitivity analysis of the effect of esketamine on intraoperative blood loss


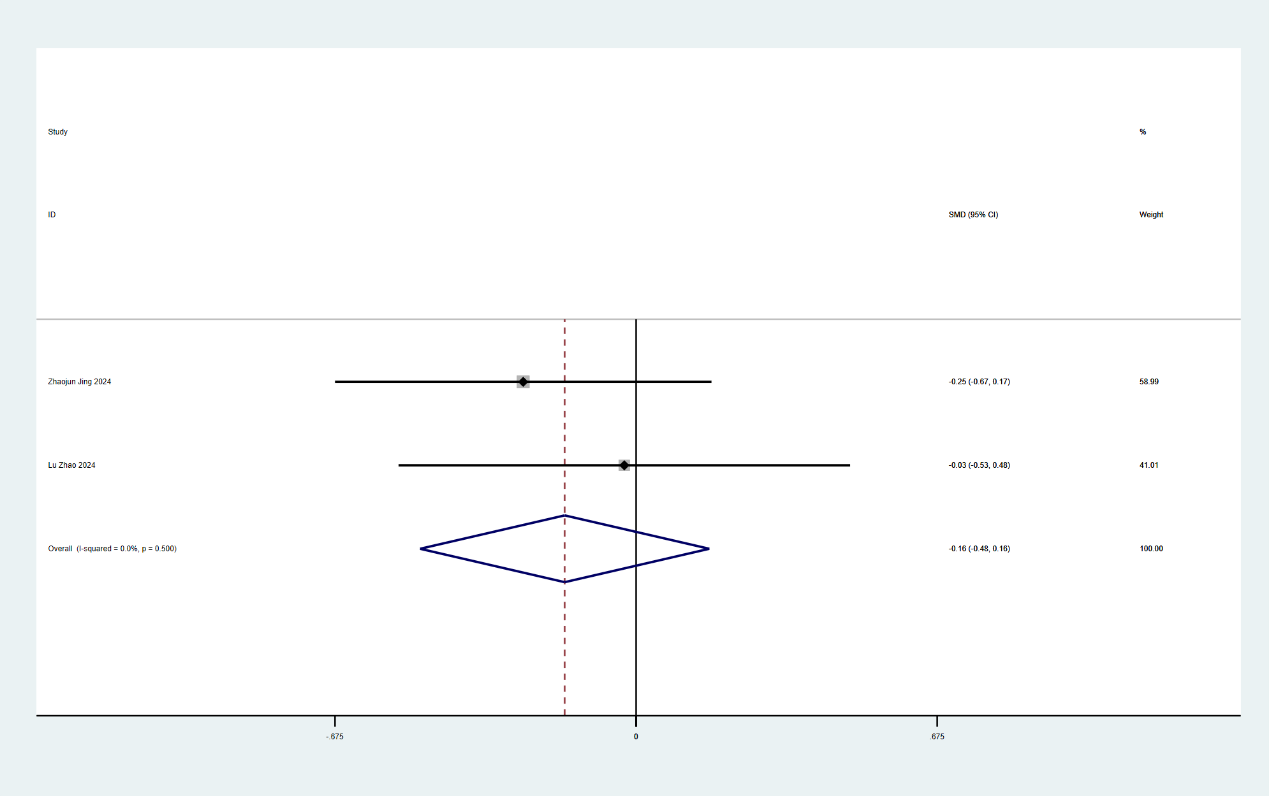


Figure S24: Forest plot of the effect of esketamine on postoperative serum IL-6


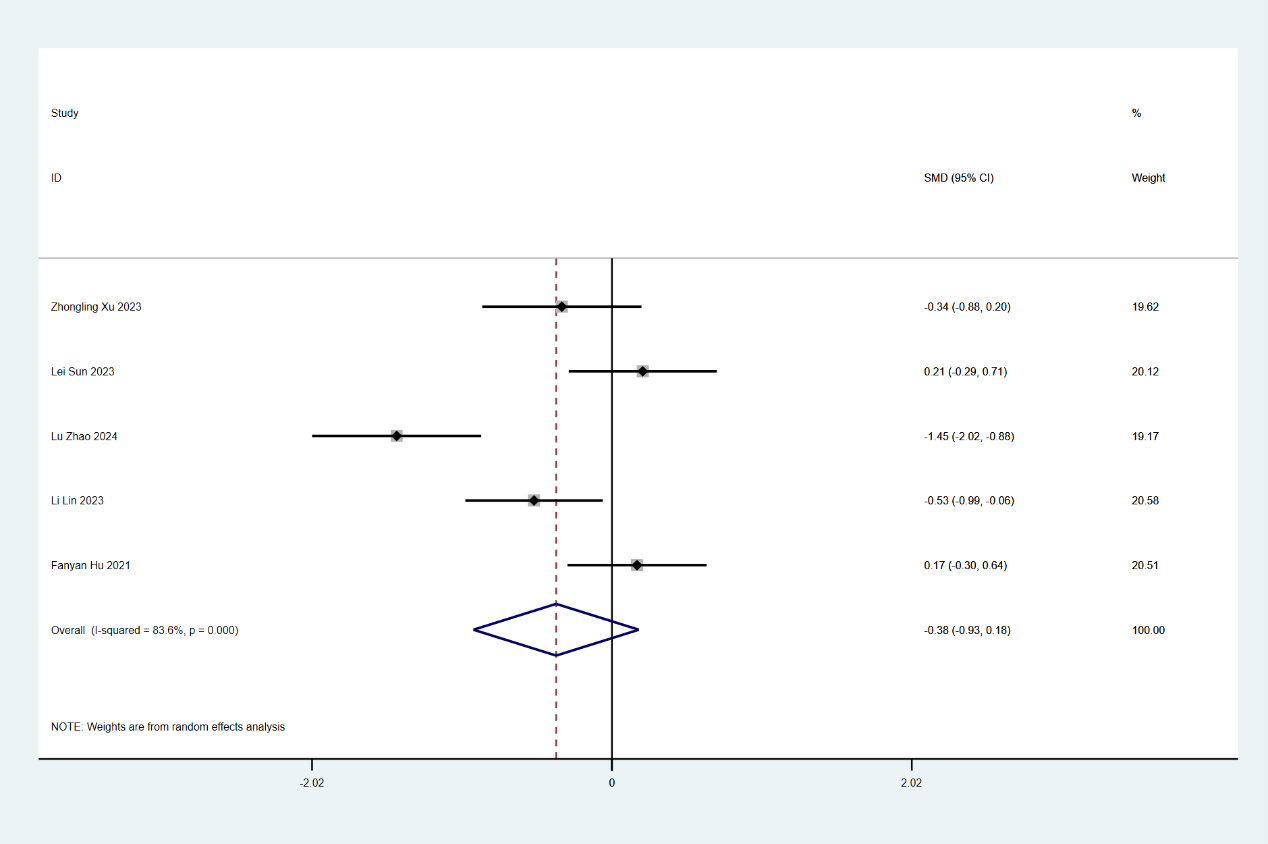


Figure S25: Forest plot of the effect of esketamine on Awakening time


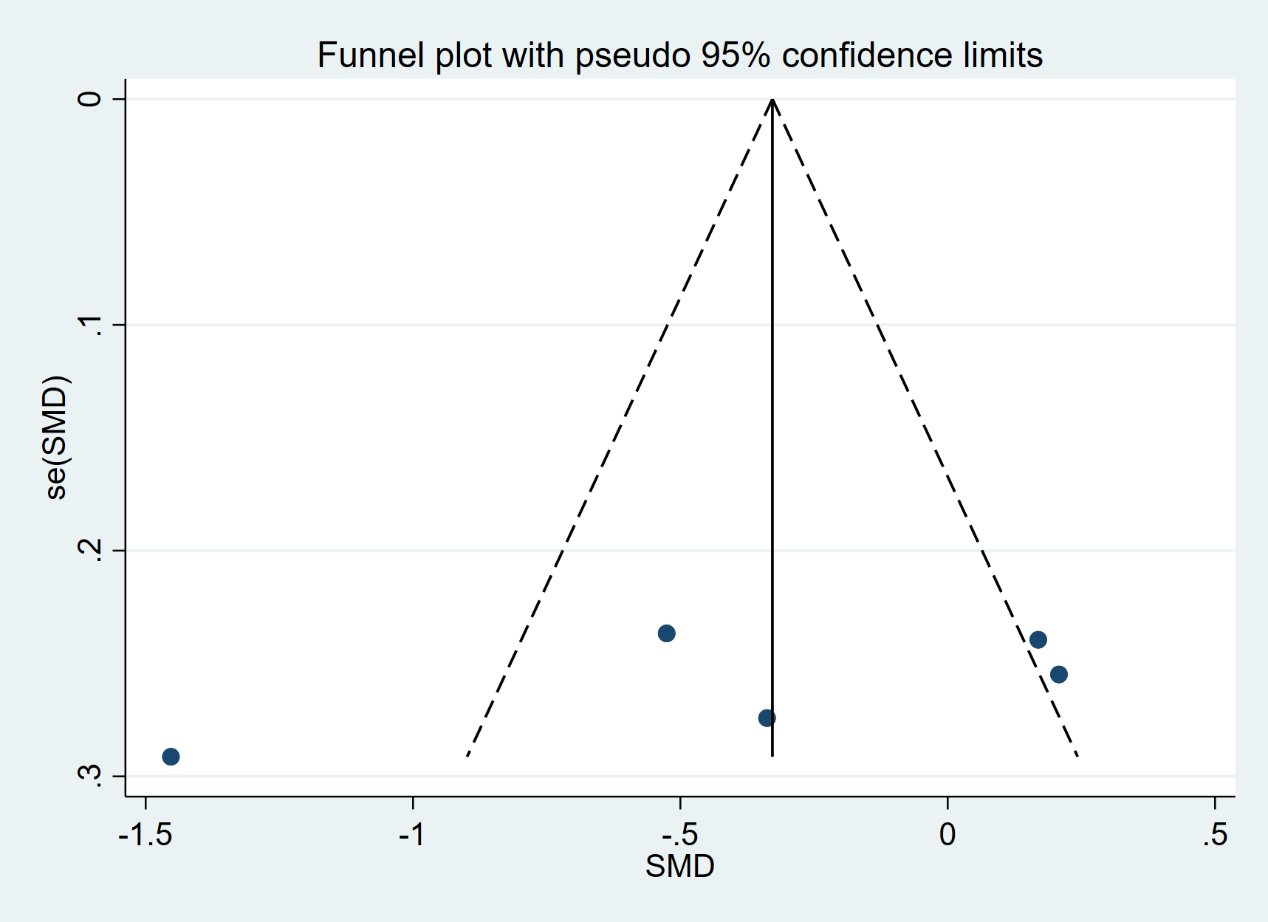


Figure S26: Funnel plot of the effect of esketamine on Awakening time


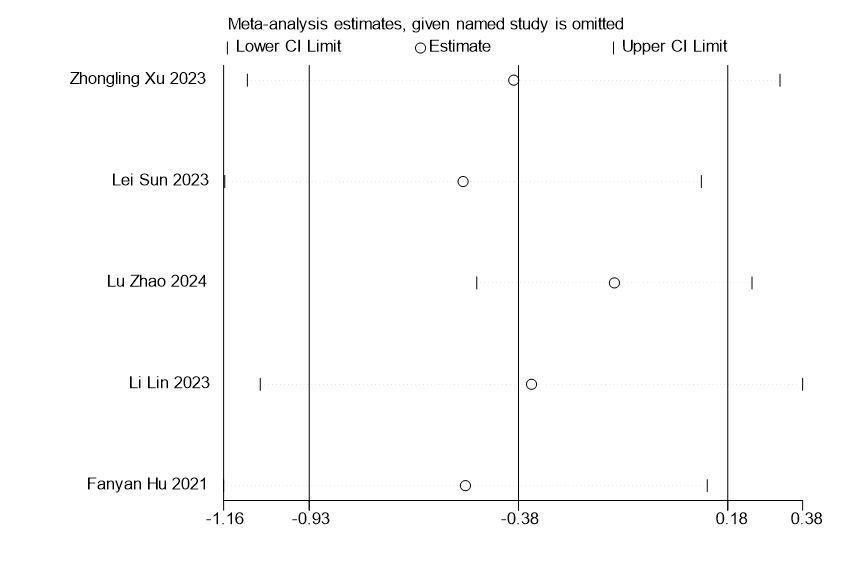


Figure S27: Sensitivity analysis of the effect of esketamine on Awakening time


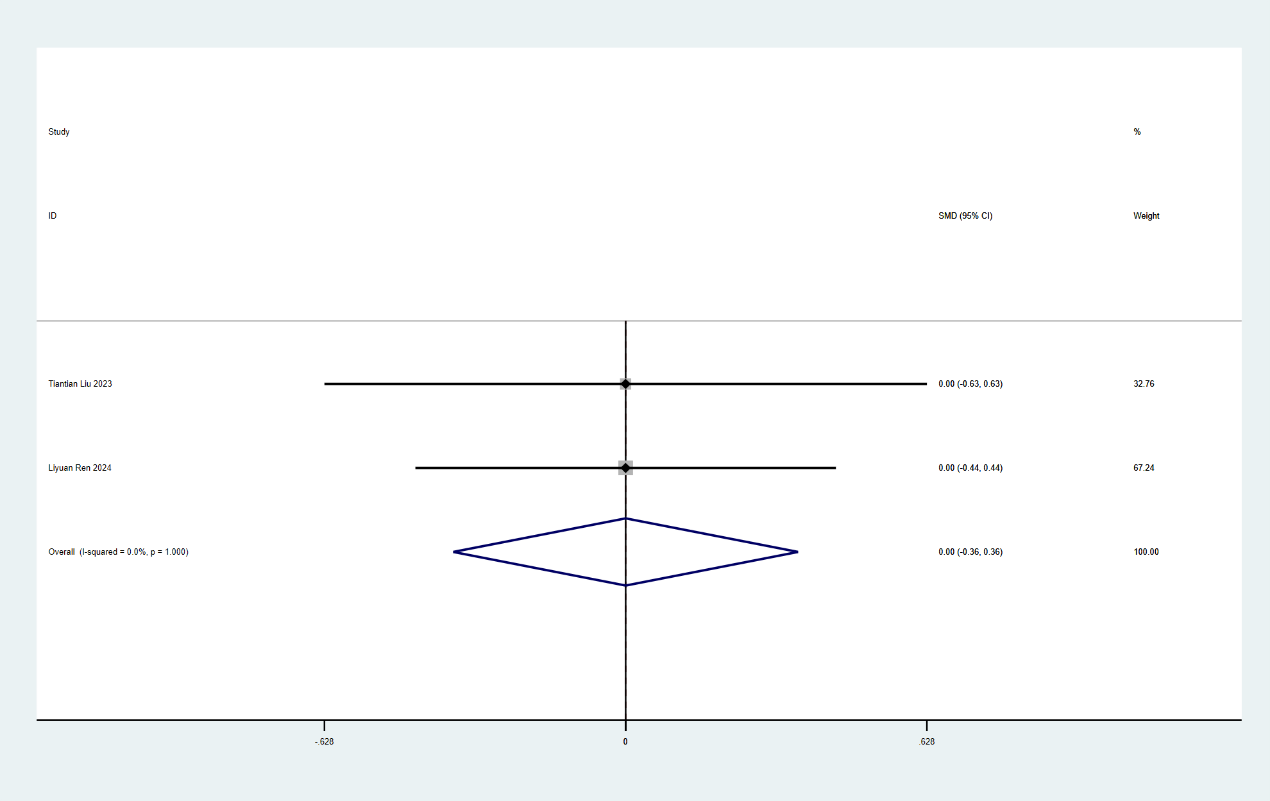


Figure S28: Forest plot of the effect of esketamine on SAS scores


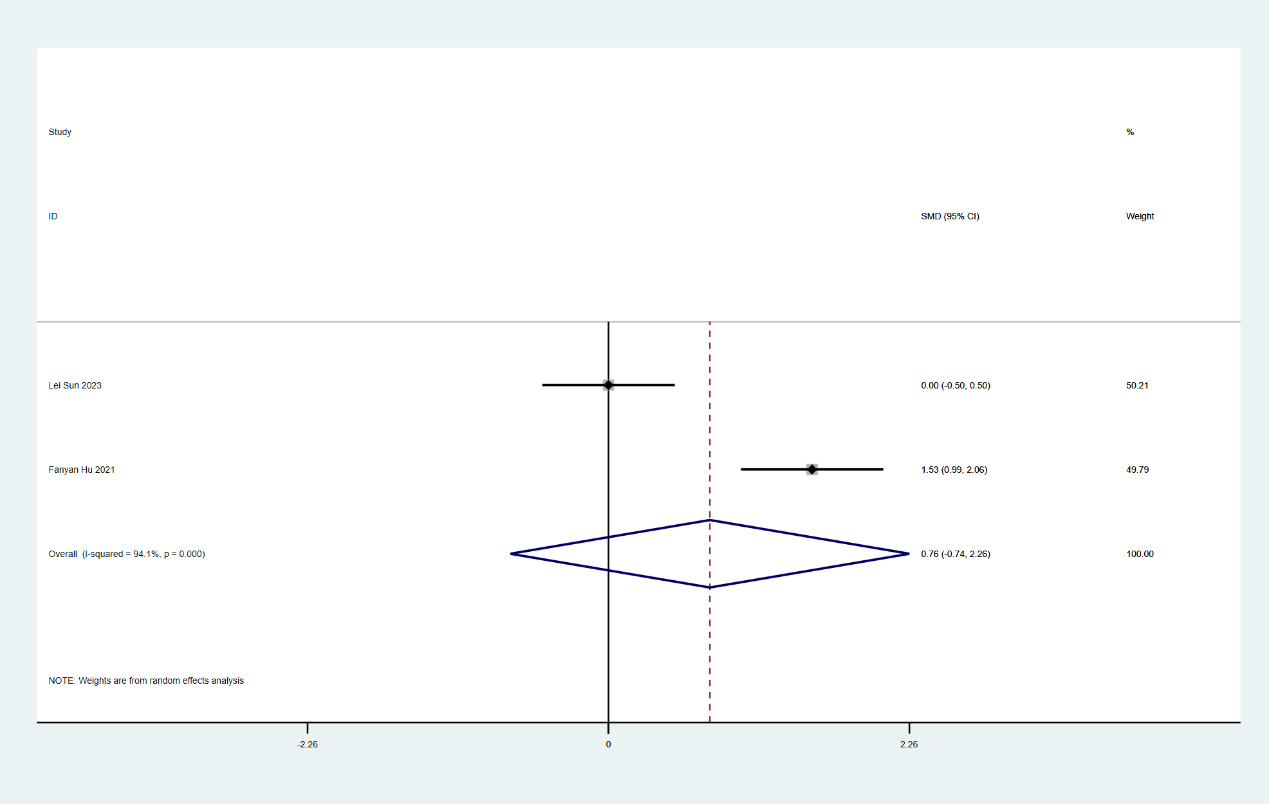


Figure S29: Forest plot of the effect of esketamine on Ramsay scores


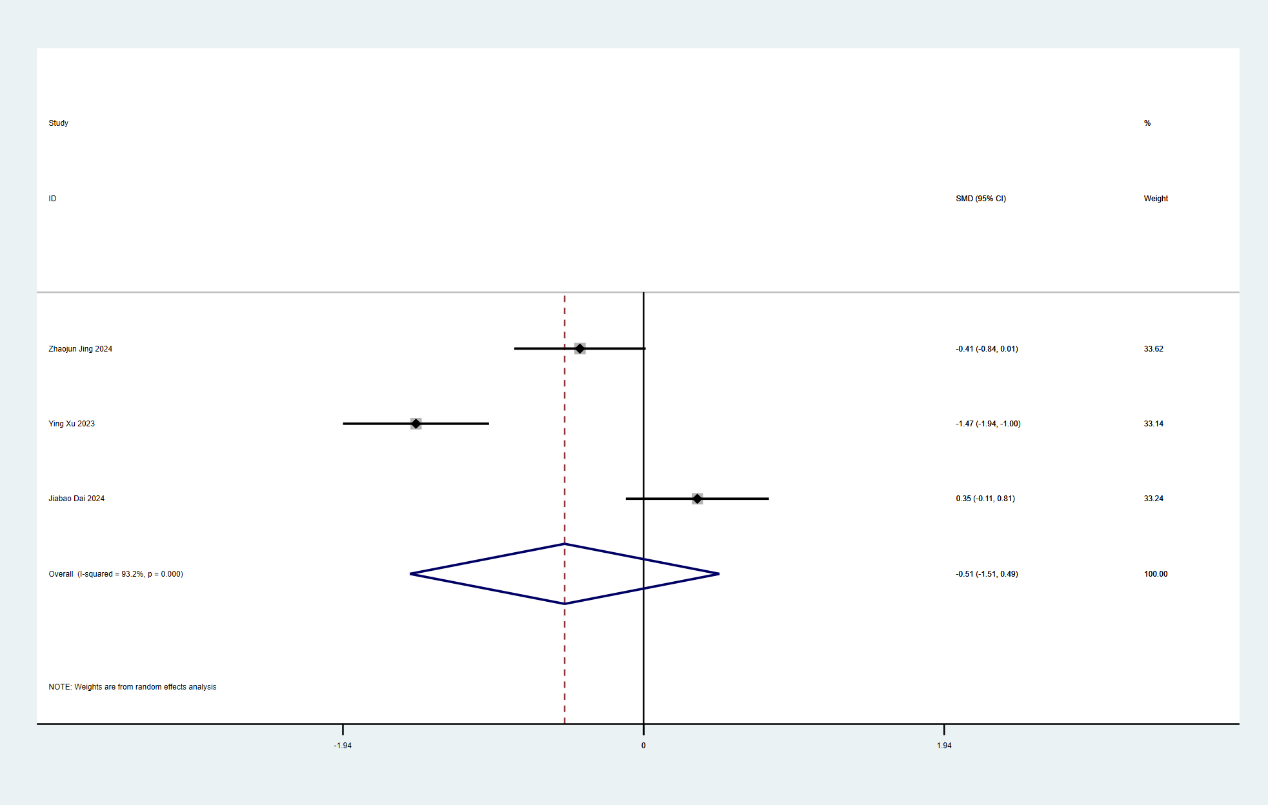


Figure S30: Forest plot of the effect of esketamine on the number of PCIA presses


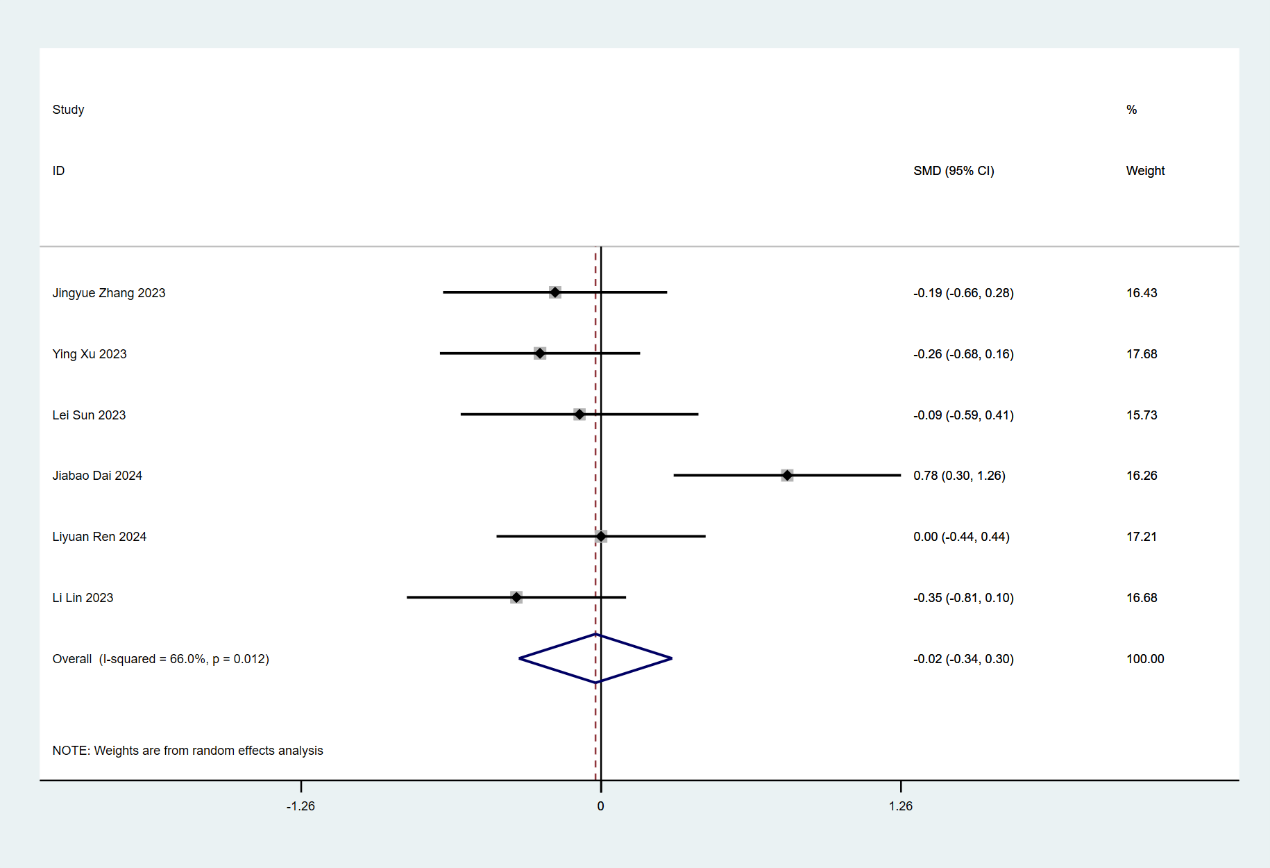


Figure S31: Forest plot of the effect of esketamine on extubation time


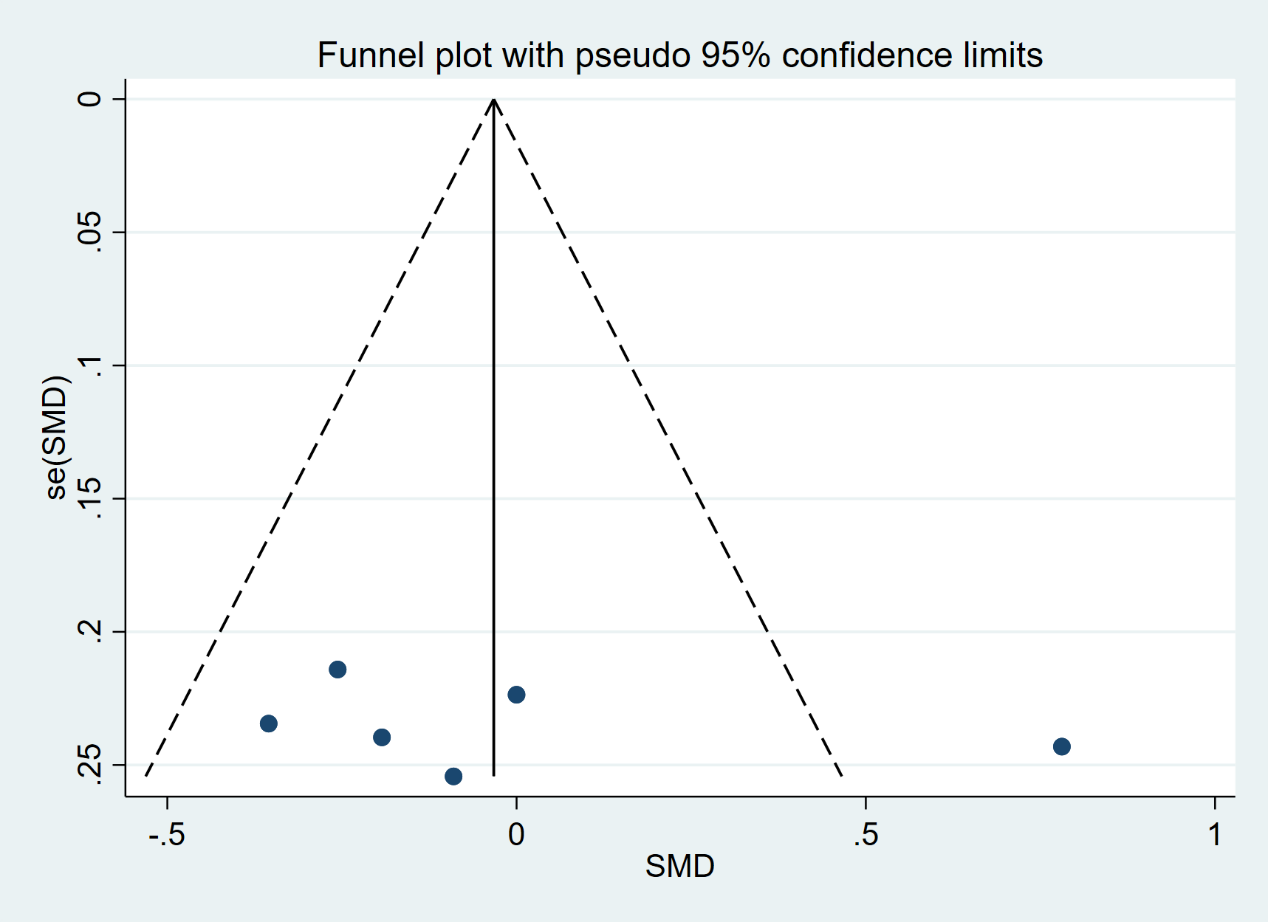


Figure S32: Funnel plot of the effect of esketamine on extubation time


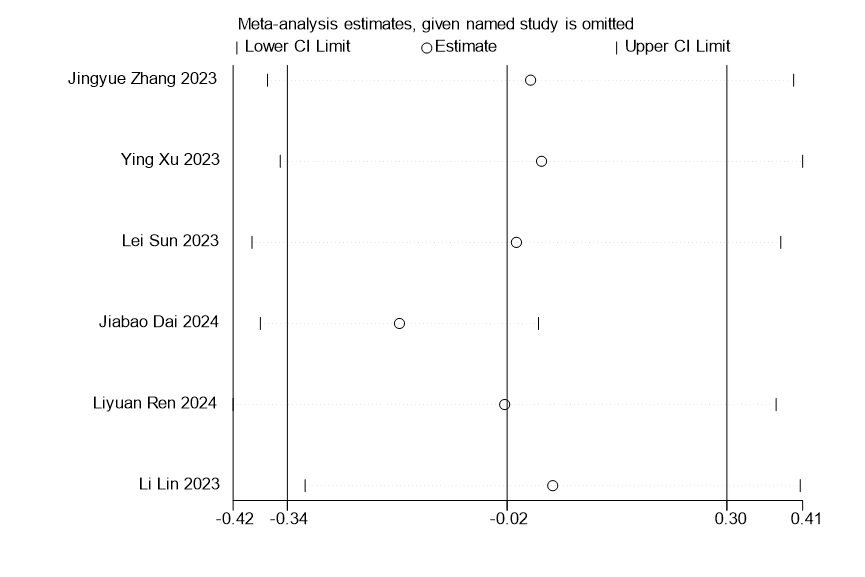


Figure S33: Sensitivity analysis of the effect of esketamine on extubation time


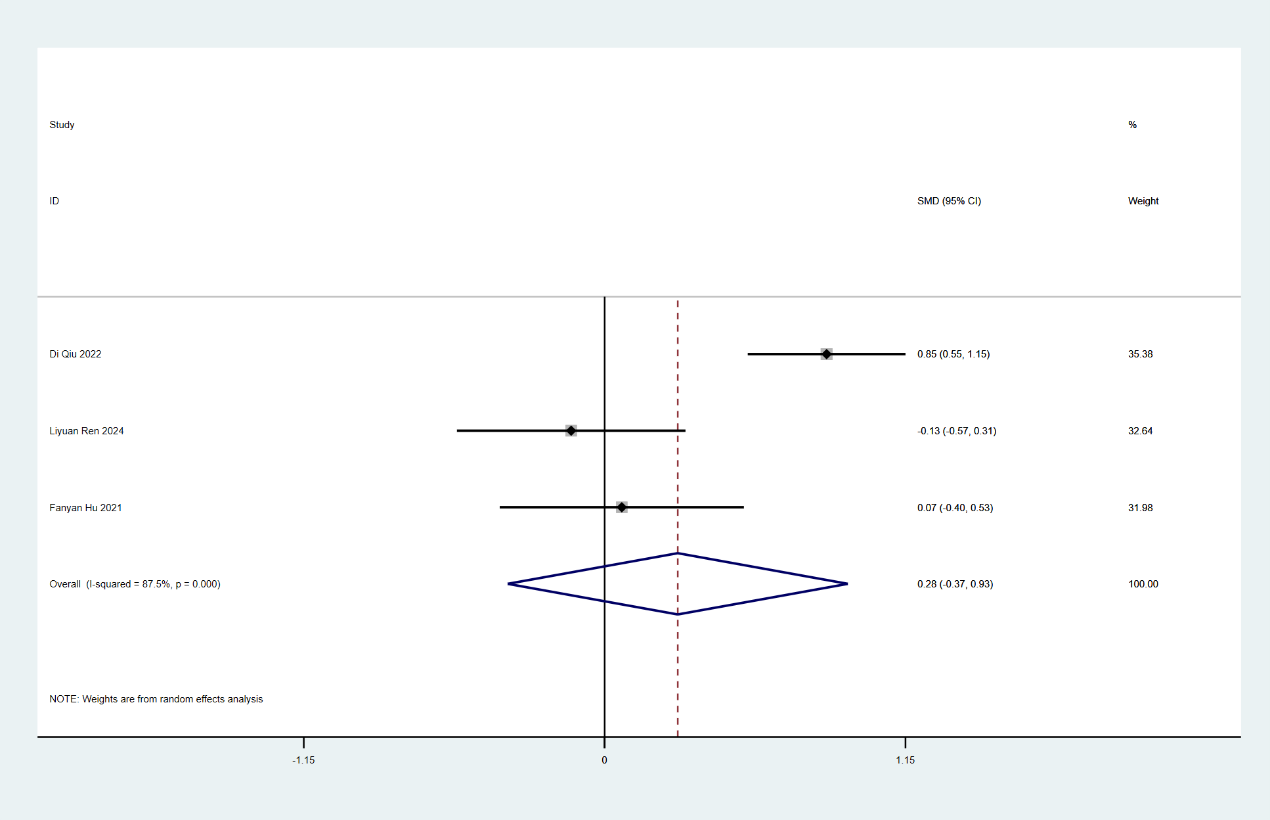


Figure S34: Forest plot of the effect of esketamine on PACU dwell times


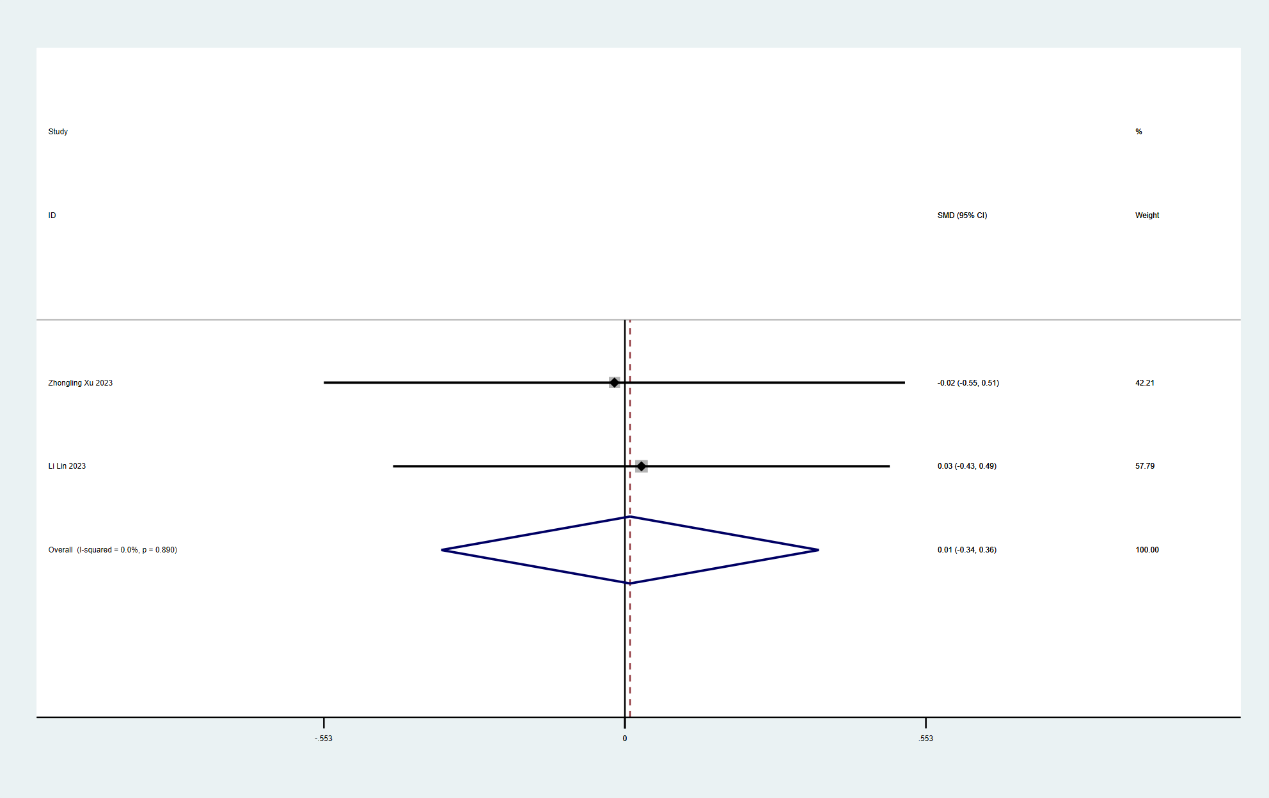


Figure S35: Forest plot of the effect of esketamine on morphine dosage


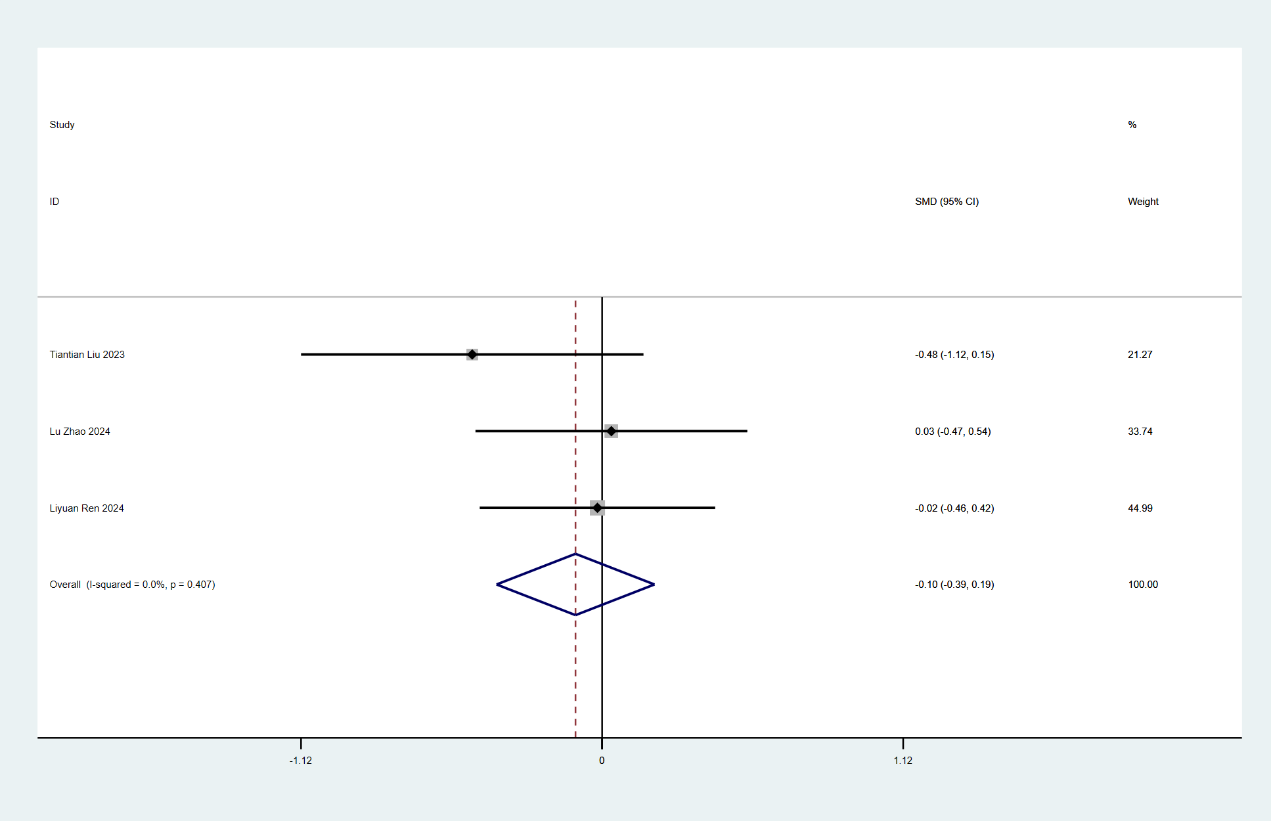


Figure S36: Forest plot of the effect of esketamine on the duration of anesthesia


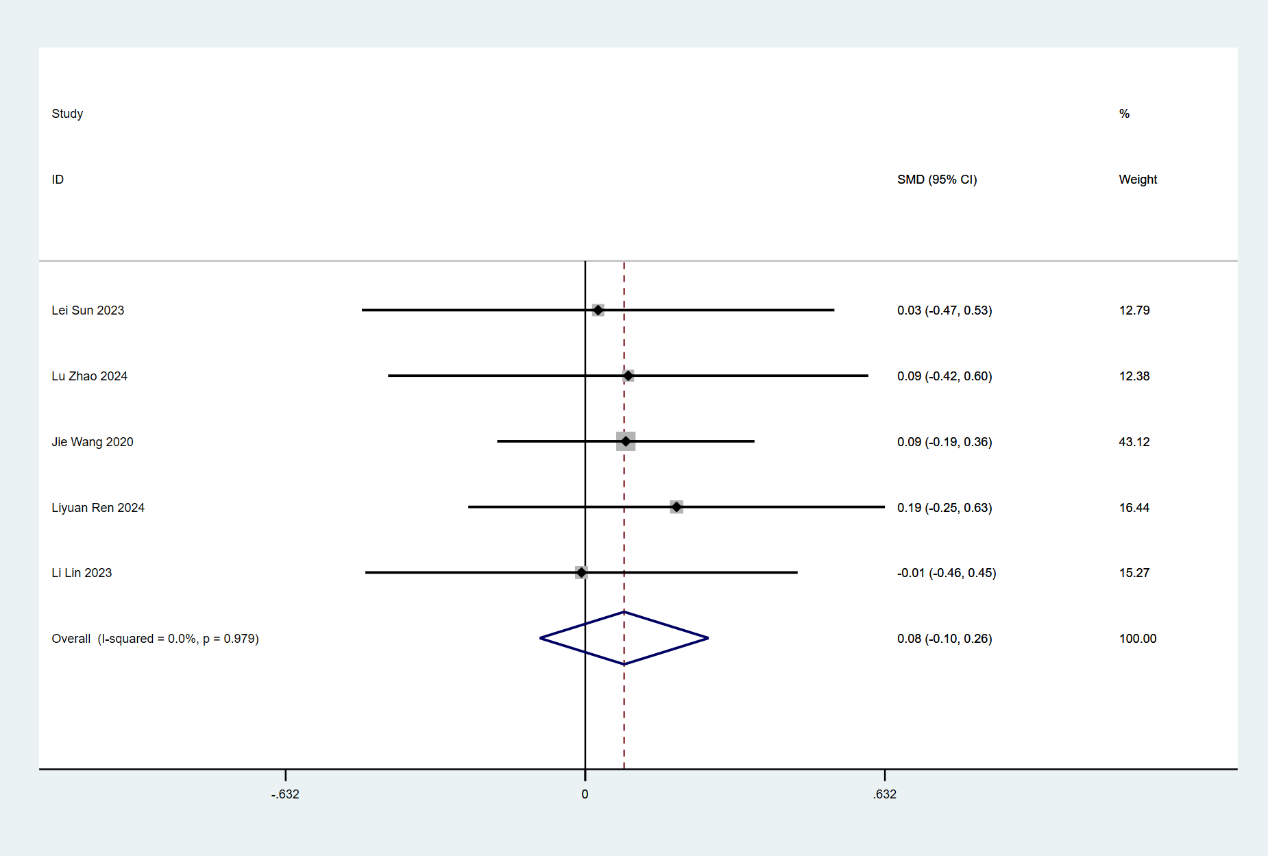


Figure S37: Forest plot of the effect of esketamine on operative time


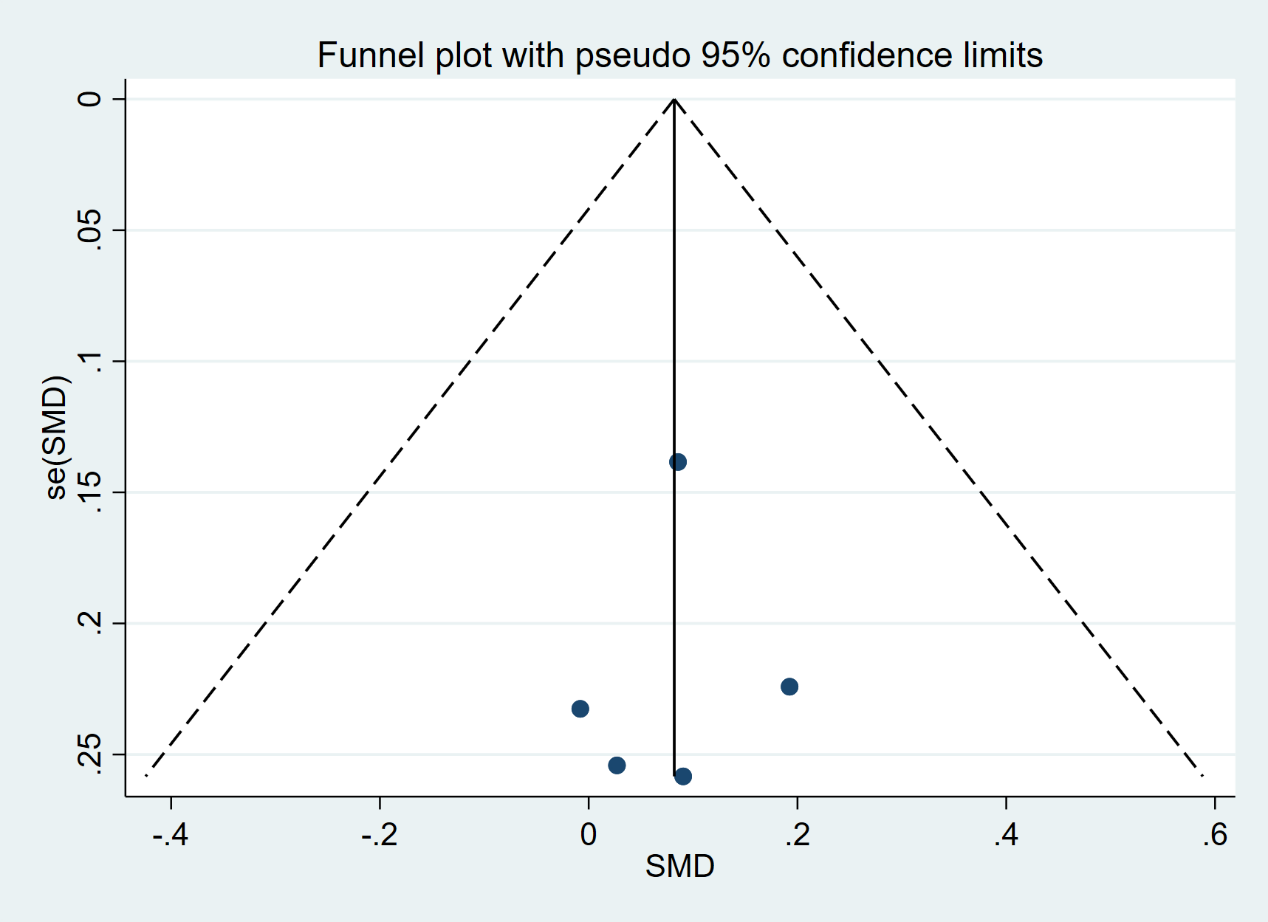


Figure S38: Funnel plot of the effect of esketamine on operative time


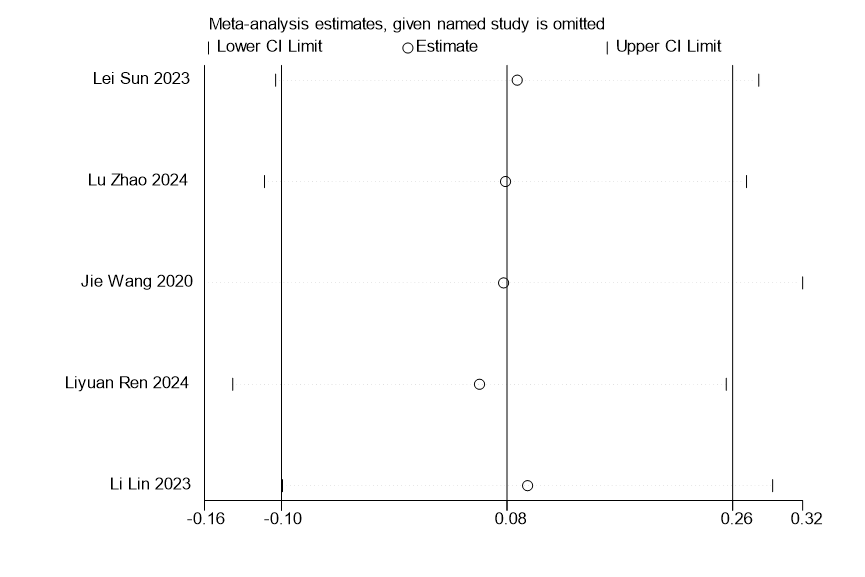


Figure S39: Sensitivity analysis of the effect of esketamine on operative time


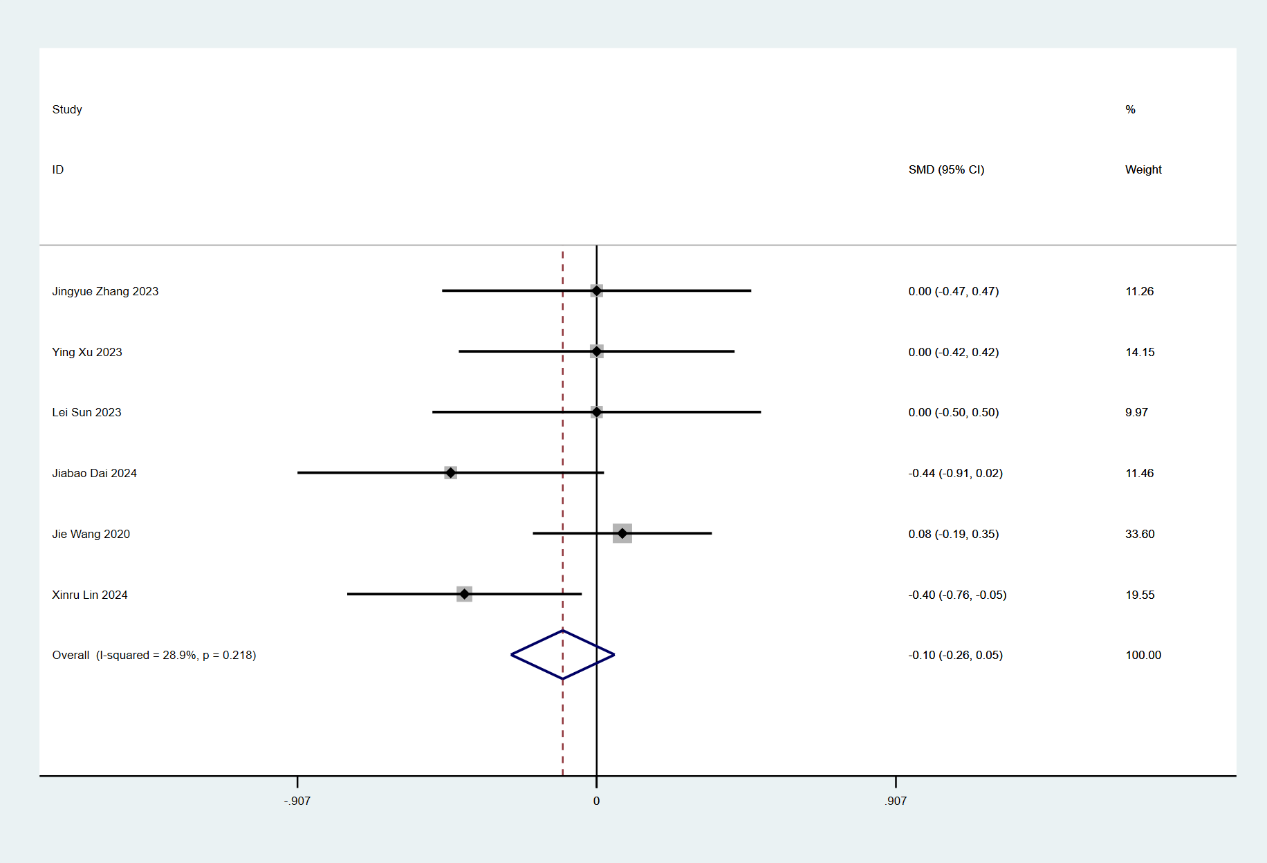


Figure S40: Forest plot of the effect of esketamine on length of hospitalization


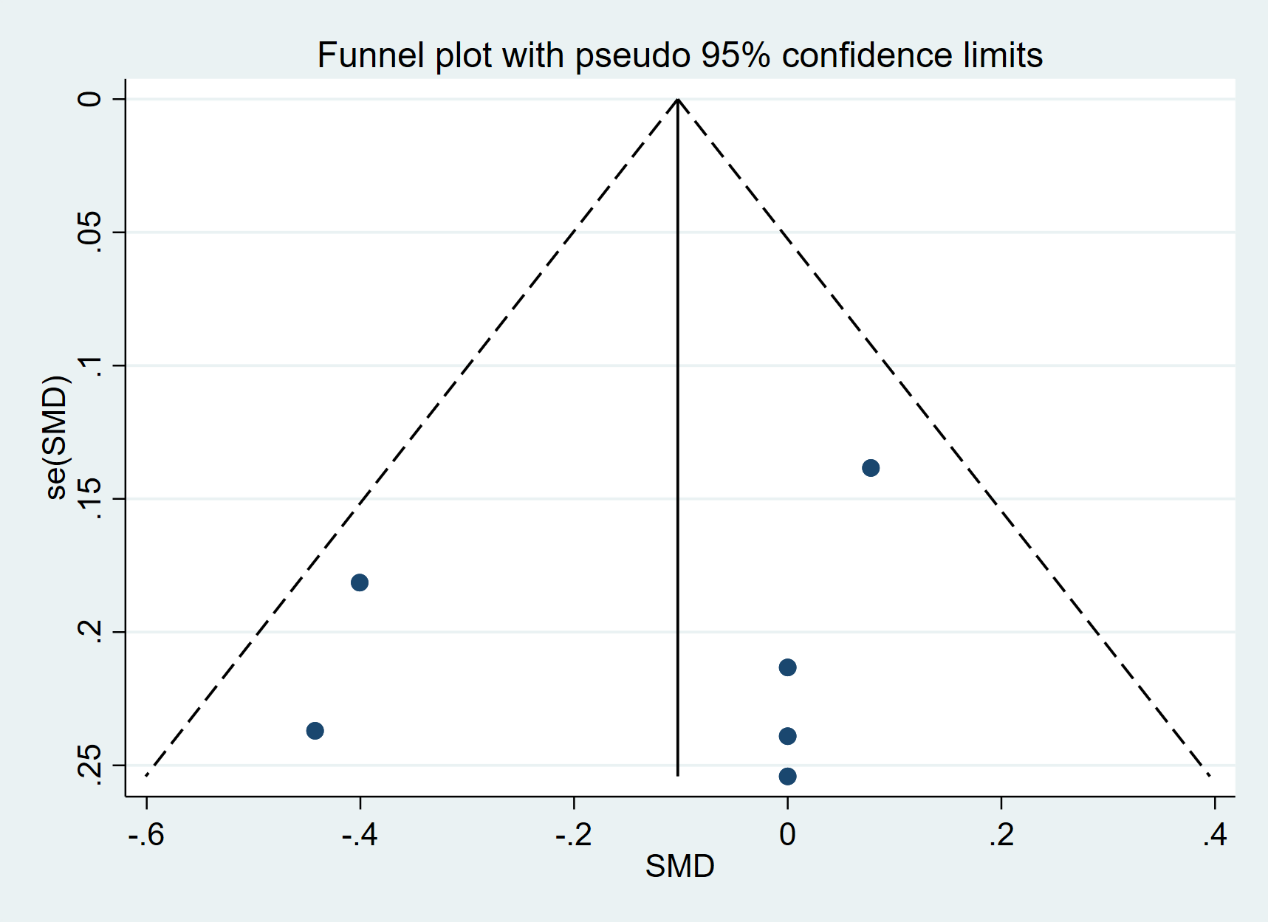


Figure S41: Funnel plot of the effect of esketamine on length of hospitalization


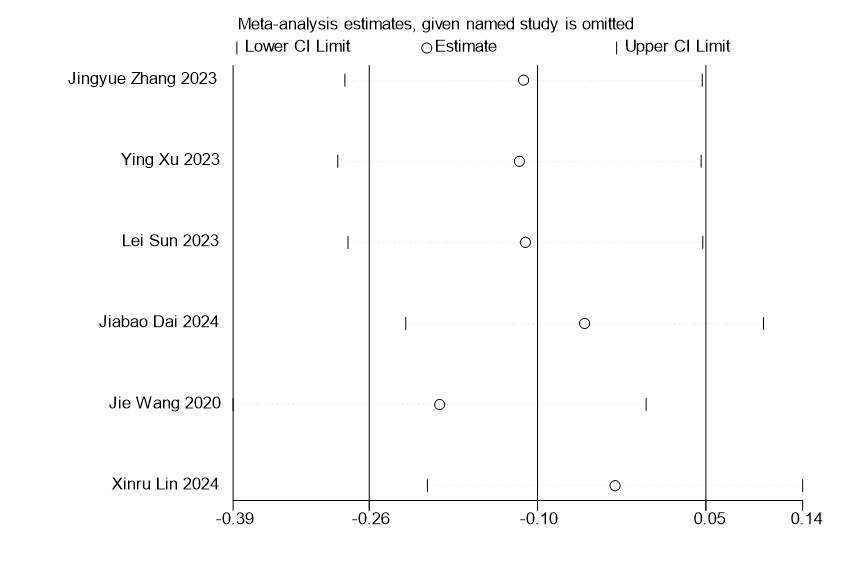


Figure S42: Sensitivity analysis of the effect of esketamine on length of hospitalization
